# Network pharmacology and UHPLC-HRMS reveal the mechanism of QSFZYL and BMSCs overexpressing IFN-γ against lung adenocarcinoma

**DOI:** 10.3389/fimmu.2025.1593121

**Published:** 2025-06-26

**Authors:** Zhen Lv, MingXuan Liu, YingYing Yang, YaHui Xie, YiHong Tian, XiangNing Xu, YinDi Wang, XingMing Wei, DongJing Ma, XueJiao Tian, JianJun Wu

**Affiliations:** School of Public Health, Gansu University of Chinese Medicine, Lanzhou, Gansu, China

**Keywords:** BMSCs, IFN-γ, JAK/STAT, UHPLC-HRMS, lung adenocarcinoma, network pharmacology

## Abstract

**Background:**

Lung cancer is a significant public health concern in China, posing a serious threat to the population. The QiShenFuZhengYiLiu (QSFZYL) is commonly prescribed as a complementary treatment for cancer patients, although its anticancer mechanism remains unclear. The purpose of this study was to explore the therapeutic mechanisms of QSFZYL in lung adenocarcinoma (LUAD).

**Methods:**

The mechanism of QSFZYL for treating LUAD was analyzed using comprehensive network pharmacology and UHPLC-HRMS, combined with experimental validation (*in vivo*).

**Results:**

Network pharmacology analysis suggested that the therapeutic effects of QSFZYL on LUAD may involve the JAK/STAT signaling pathway. UHPLC-HRMS identified 26 differential components, with representative compounds including astragalus lysine alkaloids, monoterpenoids, isoflavonoids, and flavonoids. *In vivo* experiments demonstrated that QSFZYL combined with IFN-γ significantly inhibited LUAD growth and promoted infiltration of CD3 and CD8 T cell, and downregulated JAK2, STAT3, and PD-L1 expression, promoted apoptosis.

**Conclusion:**

QSFZY combined with IFN-γ overexpressing BMSCs effectively inhibit LUAD progression. The primary mechanisms include the suppression of cancer cell growth, promotion of apoptosis and infiltration of CD3 and CD8 T cells, and inhibition of the JAK2/STAT3 signaling pathway, and downregulated PD-L1 expression.

## 1 Introduction

Lung cancer has become the leading cause of death from malignant tumors in China (1). According to global cancer statistics from 2020, there were approximately 1.79 million lung cancer-related deaths worldwide, with a case fatality rate of 18.0%, accounting for 18% of all cancer deaths globally (2). Due to the concealed nature of early-stage lung cancer, most patients present with non-specific or mild symptoms, resulting in delayed diagnosis typically at the middle or advanced stages, when survival rates are extremely low (3). Current primary treatments for lung cancer include surgery, radiotherapy, targeted therapy, and immunotherapy. However, these therapies are associated with significant side effects, such as drug resistance, liver toxicity, and renal toxicity (4, 5).

Bone marrow mesenchymal stem cells (BMSCs) have emerged as ideal vectors for tumor biotherapy due to their accessibility, ease of isolation and *in vitro* culture, and key biological properties, including exogenous gene delivery, tumor-targeting behavior, and low immunogenicity. However, highly invasive BMSCs may undergo malignant transformation within the tumor microenvironment (6). Therefore, balancing the tumorigenic potential of BMSCs with enhanced antitumor efficacy and improved vector-targeting capabilities remains a critical challenge requiring urgent attention.

Interferon-gamma (IFN-γ) is an immune cytokine secreted by CD4+ helper T cells, CD8+ cytotoxic T cells, and natural killer (NK) cells (7). It activates cellular immunity, amplifies antitumor immune responses, and directly inhibits cancer cells by inducing apoptosis and cell-cycle arrest. Additionally, IFN-γ upregulates PD-L1 expression in tumor cells, promoting immune evasion (8), while reducing STAT1 expression, which may counteract its antitumor effects (9).

Traditional Chinese medicine (TCM) is widely utilized in cancer treatment. TCM has been shown to enhance antitumor efficacy (10), alleviate clinical symptoms, and mitigate adverse reactions caused by radiotherapy, thereby improving patient quality of life (11–14). QiShenFuZhengYiLiu (QSFZYL), a modified TCM formula originally developed by Zhao Jianxiong for “fuzheng yiliu” (immune activation), incorporates Sophora flavescens Aiton and Glycyrrhiza uralensis Fisch. ex DC. to enhance synergistic effects, reducing toxicity and amplifying efficacy. Fuzheng Yiliu, when combined with IFN-γ gene-transfected BMSCs, exerts antitumor effects potentially mediated via the Bax/Bcl-2 pathway (15). An *in vitro* study further demonstrated that “fuzheng tumor-suppressing tang” reverses BMSC tumorigenicity in glioma microenvironments, inhibits telomerase activity, upregulates p53 expression, and induces apoptosis (15).

**Figure 1:**
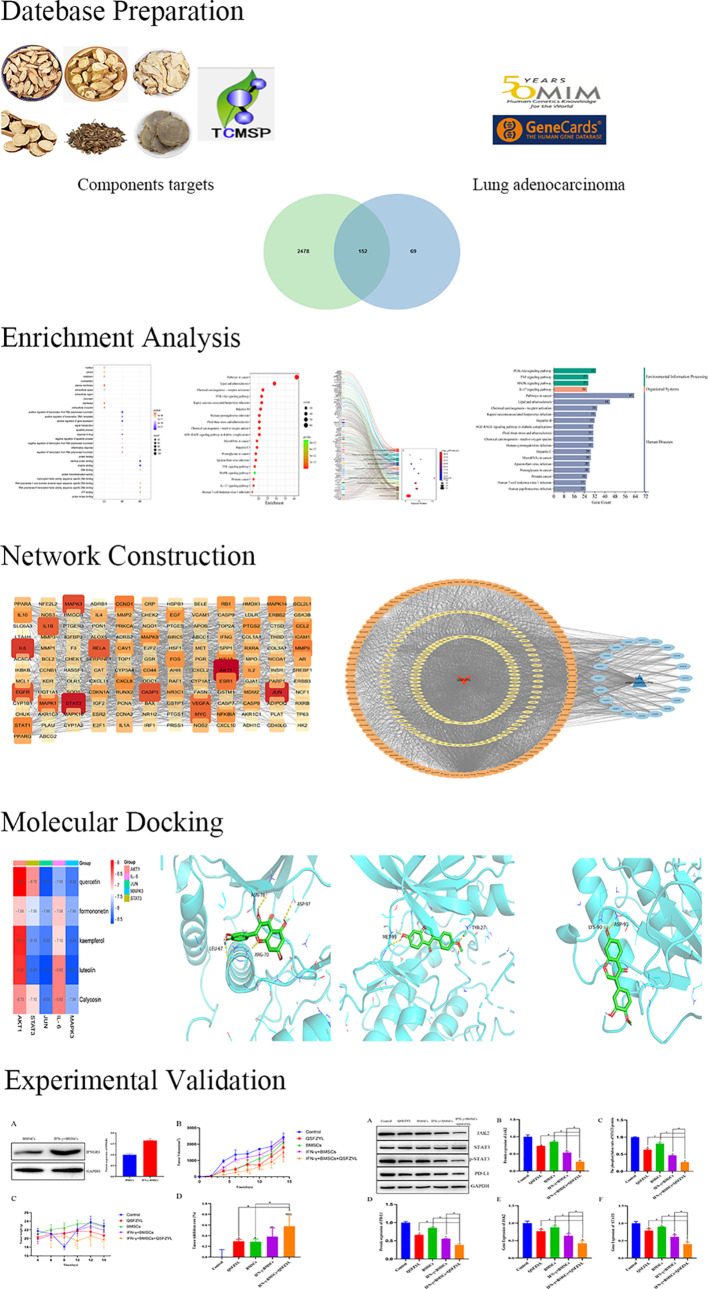
The workflow to study the molecular mechanism of QSFZYL for the treatment of lung adenocarcinoma.

Network pharmacology integrates systems biology and pharmacology to holistically evaluate the multi-component, multi-target, and multi-pathway mechanisms of herbal medicines (16, 17). Growing evidence suggests that TCM exerts therapeutic effects through targets and pathways predictable by network pharmacology (12, 18). Here, we employed network pharmacology, UHPLC-HRMS, and *in vivo* experimental validation to elucidate the mechanisms of QSFZYL combined with IFN-γ-overexpressing BMSCs in LUAD treatment. The workflow to study the molecular mechanism of QSFZYL for the treatment of lung adenocarcinoma (**Figure 1**).

## 2 Materials and methods

### 2.1 Cells and experimental animals

Lewis cells were obtained from the Cell Bank of the Chinese Academy of Sciences. The cells were cultured in high-glucose Dulbecco’s Modified Eagle Medium (DMEM; Corning Corp., Corning, NY, USA) supplemented with 10% fetal bovine serum (FBS; Gibco, Grand Island, NY, USA) and 1% penicillin/streptomycin (Baiyutai Corp., China); the medium was changed every 2 days.

A total of 30 C57BL/6 mice (male, weighing 20–25 g, aged 4–6 weeks) were provided by Spectrum Biotechnology Co. Ltd. (Beijing, China; license no. SCXK [Beijing] 2019-0010). All animals were housed individually in a pathogen-free facility under a 12-h dark/light cycle, fed a standard laboratory diet, and maintained at a temperature of 23–25°C and humidity-controlled (45-55%). Prior to the experiment, the mice underwent a 1-week acclimatisation period in the animal facility. The study protocol was approved by the Laboratory Animal Committee of Gansu University of Traditional Chinese Medicine (no. SY2023-622).

### 2.2 Preparation of drugs

The raw materials used to prepare QSFZYL were the rhizomes of commonly used Chinese herbs radix hedysari (*Hedysarum polybotrys* Hand.-Mazz.), radix angelicae sinensis (*Angelica sinensis* (Oliv.) Diels), rhizoma curcumae (*Curcuma phaeocaulis* Valeton), radix polygoni multiflora (*Patrinia heterophylla* Bunge), radix sophorae flavescentis (*S. flavescens* Aiton), and radix glycyrrhizae (*G. uralensis* Fisch. ex DC.) (**Table 1**), which are roots and stems derived from plants. And all herbs were obtained from the Affiliated Hospital of Gansu University of Traditional Chinese Medicine. These herbs were soaked in pure water for 40 min in a ratio of 3:1:1:3:1:1, and then the mixture was boiled and decocted at normal pressure for 1.5 h. The residue was filtered, fresh water was added, and the decoction was boiled again and simmered for 1 h. The second residue was filtered and the two filtrates were combined and concentrated to yield a solution containing 2 g of crude drug per mL, which was stored at −4°C for later use.

**Table 1:** Main components of QSFZYL.

Component	Latin name	Component used	Proportion
Hedysarum polybotrys Hand. (Hongqi)	*Hedysarum polybotrys* Hand.-Mazz.	Rhizome	3
Patrinia heterophylla (Mutouhui)	*Patrinia heterophylla* Bunge	Rhizome	3
Curcuma aeruginosa Roxb. (Ezhu)	*Curcuma phaeocaulis* Valeton	Rhizome	1
Angelica inensis (Danggui)	*Angelica sinensis* (Oliv.) Diels	Rhizome	1
Sophora flavescens Ait. (Kusheng)	*Sophora flavescens* Aiton	Rhizome	1
Liquorice (Gancao)	*Glycyrrhiza uralensis* Fisch. ex DC.	Rhizome	1

### 2.3 Target prediction of QSFZYL and LUAD

The chemical components of QSFZYL were retrieved from the Traditional Chinese Medicine Systems Pharmacology (TCMSP) database and selected based on the criteria oral bioavailability ≥ 30% and drug-likeness ≥ 0.18. The corresponding targets of these components were predicted, and all collected targets were standardized using the UniProt database to obtain the relevant gene names. These target genes were imported into Cytoscape v3.10.0 for network analysis to identify key active ingredients in QSFZYL.

For LUAD, targets were retrieved from the GeneCards (https://www.genecards.org/) and OMIM (https://omim.org/) databases.

A Venn diagram was generated to visualize the intersection of drug targets and disease-associated genes. The overlapping genes, along with drug ingredients and LUAD targets, were integrated into Cytoscape. Degree values were calculated to prioritize core active constituents of QSFZYL.

### 2.4 Enrichment analysis of key targets

Key targets were uploaded to the Metascape database (http://metascape.org) for functional enrichment analysis. Kyoto Encyclopedia of Genes and Genomes (KEGG) and Gene Ontology (GO) pathway analyses were performed using the Bioinformatics Cloud Platform (http://www.bioinformatics.com.cn). A target pathway network was constructed in Cytoscape to visualize interactions between active ingredients and pathways.

### 2.5 Protein–protein interaction network construction

Intersection genes were imported into the STRING database (https://string-db.org/) with an interaction confidence score threshold of 0.700. Filtered PPI data were visualized in Cytoscape. Top 10 hub genes were selected based on degree centrality and subgraph density using the CytoHubba plugin. A protein interaction network diagram was generated to highlight critical targets.

### 2.6 Molecular docking analysis

Core target structures were downloaded from the PubChem (https://pubchem.ncbi.nlm.nih.gov/) and PDB (https://www.rcsb.org/) databases. Protein structures were optimized for binding energy minimization using Chem3D. The receptor was prepared in PyMOL, hydrogenated via AutoDockTools, and docked with ligands using AutoDock Vina. Binding poses were visualized and analyzed in PyMol.

### 2.7 Preparation of samples

#### 2.7.1 Extraction of QSFZYL samples

Transfer 600 μL of the sample into a 1.5 mL Eppendorf tube, add 400 μL of pure methanol, vortex mix for 10 seconds, and collect 200 μL of the supernatant. Add 200 μL of 40% (v/v) methanol aqueous solution to the aliquot, vortex mix for 10 seconds, centrifuge at 16,000 g and 4°C for 15 minutes, and retain the supernatant for further analysis.

#### 2.7.2 Extraction of serum samples

Aliquot an appropriate volume of serum, add methanol (1:1 volume ratio), vortex mix for 60 seconds, and incubate at -20°C for 30 minutes. Centrifuge at 16,000 × g and 4°C for 20 minutes, collect the supernatant, and vacuum-dry the residue. Reconstitute the residue with 100 μL of 40% (v/v) methanol aqueous solution, vortex mix, centrifuge at 16,000 g and 4°C for 15 minutes, and collect the final supernatant.

#### 2.7.3 Extraction of plasma+QSFZYL samples

Combine plasma with QSFZYL extraction buffer (1:1 volume ratio), add methanol (1:1 volume ratio), vortex mix for 60 seconds, and incubate at -20°C for 30 minutes. Centrifuge at 16,000 g and 4°C for 20 minutes, collect the supernatant, and vacuum-dry the residue. Reconstitute the residue with 100 μL of 40% (v/v) methanol aqueous solution, vortex mix, centrifuge at 16,000 g and 4°C for 15 minutes, and collect the supernatant as the final extract.

### 2.8 Chromatography-mass spectrometry conditions

#### 2.8.1 UHPLC parameters

Analyses were performed on a Vanquish UHPLC system (Thermo Fisher Scientific, Bremen, Germany) coupled with an ACQUITY UPLC HSS T3 column (2.1 mm × 100 mm, 1.8 μm). The mobile phase comprised (A) 0.1% formic acid in water and (B) 0.1% formic acid in acetonitrile, delivered at 0.3 mL/min with a gradient elution program.

#### 2.8.2 MS parameters​

Mass spectrometric data were acquired using a Q-Exactive HFX mass spectrometer (Thermo Fisher Scientific) in electrospray ionization (ESI) positive and negative modes. Source settings included a capillary voltage of 3800 V (ESI^+^)/3500 V (ESI^-^), sheath gas pressure of 45 arb, auxiliary gas pressure of 20 arb, ion transfer tube temperature of 320°C, and nebulizer temperature of 350°C. Data were acquired in full-scan/dd-MS² mode (resolution: 60,000 and 15,000, respectively) with a mass range of *m/z* 90–1300. Collision energies were stepwise 20, 40, and 60 eV for top 10 precursor ions.

#### 2.8.3 Sample analysis

Samples (6 μL each of blank, dosing, and blank+QSFZYL groups; 2 μL of QSFZYL solution) were injected in triplicate into the LC-MS system. The injection sequence included one run for blank and dosing groups, followed by five repeated injections for blank+QSFZYL and QSFZYL groups to ensure reproducibility.

### 2.9 Primary culture, isolation, and identification of BMSCs

C57BL/6 mice (SPF grade, aged 2–3 weeks) were sacrificed and soaked in 75% alcohol for 30 min. Femurs and tibias were separated, cut in the middle and flushed with serum-free DMEM-H culture solution until the marrow cavity turned white. The fluid was collected, filtered using a 70-μm cell strainer. The cell suspension was centrifuged (1,000 g, 5 min), resuspended in red blood cell lysis buffer, and centrifuged again (1,200 g, 5 min). After washing with PBS, cells were cultured in DMEM-H supplemented with 15% FBS, 100 U/mL penicillin, and 100 U/mL streptomycin. Non-adherent cells were removed after 48 h, and adherent BMSCs were passaged at a 1:2 ratio every 2 days. BMSCs from passage 5 were used for subsequent experiments. Surface antigen analysis was performed at passage 3, and the molecular markers CD44, CD105, CD34, and CD45 were detected by flow cytometry.

### 2.10 IFN-γ expression lentiviral vector construction

BMSCs (passage 3, 4 × 10^4^ cells/well) were seeded in 6-well plates. Lentivirus (MOI = 20; titer = 2.5 × 10^8^ TU/mL) was added in 2 mL DMEM-H per well, followed by transduction at 37°C (5% CO_2_). After 24 h of cell culture, the culture medium was replaced. Virus expression was observed 48 h after infection, and Western blotting was performed.

### 2.11 C57BL/6 mouse models

Lewis tumor cells (10^6^ cells/200 μL) were subcutaneously injected into the right axilla of mice under aseptic conditions. The injection site was disinfected with alcohol swabs, and cells were delivered using a 25G needle to minimize leakage.

### 2.12 Grouping and treatment of C57BL/6 mice

Tumor-bearing mice (n = 30) were randomized into five groups (n = 6/group): Control, BMSCs, QSFZYL, IFN-γ + BMSCs, and IFN-γ + BMSCs + QSFZYL. The BMSC group received tail vein injections of BMSC suspension (5 × 10^4^ cells/mL, 0.2 mL) five times, with 2-day intervals between injections. The QSFZYL and IFN-γ + BMSC + QSFZYL groups were treated with QSFZYL (0.32 mL) once daily. The IFN-γ + BMSC and IFN-γ + BMSC + QSFZYL groups received tail vein injections of BMSC lentiviral cell suspension (5 × 10^4^ cells/mL, 0.2 mL) five times, with 2-day intervals between each injection.

Humane endpoints followed AVMA guidelines: body weight loss >20%, inability to ambulate, or tumor volume ≥2 cm³. Mice were euthanized via sodium pentobarbital (50 mg/kg, i.p.), and tumors were snap-frozen (-80°C). All procedures complied with ARRIVE guidelines.

### 2.13 Tumor growth in mice

Observation of the inoculation site began on day 4 post-inoculation. Once a tumor was detected, its size was promptly recorded. The length and width of the subcutaneous tumor were measured on alternate days, and the tumor volume was calculated as follows:


$$\text{Tumor volume}=\text{L}\times {\text{W}}^{2}/2$$

where L is the average tumor length and W is the average tumor width.

After the mice were euthanised, the tumor tissue was weighed, and the tumor growth inhibition rate was calculated as follows:


Tumor  growth  inhibition  rate(%)=(1–T/C)×100%

where T is the average tumor weight in the treatment group, and C is the average tumor weight in the control group.

### 2.14 Western blotting to detect JAK2, p-STAT3, STAT 3, and PD-L1 protein expression in tumor tissues

Tumor tissues from tumor-bearing mice in each group were ground using a cryogrinder. Radioimmunoprecipitation assay lysis buffer containing phosphatase inhibitors and phenylmethylsulfonyl fluoride was added to extract total protein, and protein concentration was determined using a bicinchoninic acid assay. Protein samples were subjected to 10% sodium dodecyl sulfate–polyacrylamide gel electrophoresis, transferred to a polyvinylidene fluoride membrane and blocked with 5% skim milk at room temperature for 2 h. The membranes were incubated overnight at 4°C with the respective primary antibodies, washed with PBS and Tween 20 (PBST), and then incubated with secondary antibodies for 2 h. After washing three times with PBST, the proteins were visualized using an electrochemiluminescence reagent and analyzed with ImageJ software. The primary antibodies used included STAT3 (Abcam, Cambridge, UK), p-STAT3 (Solarbio, Wuhan, China), JAK2 (Solarbio), PD-L1 (Servicebio Technology, Wuhan, China), GAPDH (Huabio, Woburn, MA, USA) and the secondary antibody is goat anti-rabbit IgG-HRP(Absin Bioscience Inc., USA). The specific information is shown in **Table 2**.

**Table 2:** Internal reference and target gene primers.

Gene	Nucleotide sequence	Amplification length (bp)	Login ID
GAPDH-F	TGTTTCCTCGTCCCGTAG	108	NM_001048177.3
GAPDH-R	CAATCTCCACTTTGCCACT		
JAK2-F	GCAACCTCCACATCTCCTGT	125	NM_011486.5
JAK2-R	TACTCTCCTTCAGCTTGCCC		
STAT3-F	TTGGAAAGTACTGTAGGCCCG	216	NM_001289726.2
STAT3-R	TCACATGGGGGAGGTAGCAC		

### 2.15 Quantitative reverse-transcription polymerase chain reaction to detect JAK2 and STAT3 gene expression in tumor tissues

Mouse tumor tissues from each group were collected, and total RNA was extracted using Trizol reagent. Complementary DNA was synthesized from the extracted RNA, and qRT-PCR was performed using complementary DNA as the template. Primers were designed and synthesized by Biomed Bioengineering Co., Ltd. (Beijing, China). The qRT-PCR reaction parameters were as follows: pre-denaturation at 95°C for 2 min, followed by denaturation at 95°C for 10 s, annealing and extension at 60°C for 30 s, for a total of 40 cycles. Relative gene expression levels were calculated using the 2^-ΔΔCt^ method, with GAPDH as the internal reference. Primers for both reference and target genes were designed based on gene accession numbers available in the National Center for Biotechnology Information database.

### 2.16 Immunofluorescent staining

Paraffin-embedded specimens were sectioned at 4-mm thickness. The antigen retrieval was applied in a pressure cooker for 30min with the citrate buffer (pH 6.0), and they were blocked in PBS containing with 10% goat serum for 60 min at 37°C. After that, the sections were incubated with antibodies specific for rabbit-anti-mouse CD3 and CD8 (Bioss, Beijing, China) with an enveloping fluorescent probe overnight at 4°C. DAPI (Solarbio, Shanghai, China) was then used to counterstain the nuclei and images were obtained by fluorescence microscope.

### 2.17 TUNEL assay for tumor cell apoptosis

The TUNEL assay was performed according to the kit instructions, and apoptotic cells were observed and photographed under a fluorescence microscope.

### 2.18 Statistical analysis

Statistical analyses were performed using GraphPad Prism v8.0 (GraphPad Software Inc., San Diego, CA, USA). Data are expressed as means ± standard deviation (SD). Differences between each groups were assessed using one-way analysis of variance (ANOVA) followed by Tukey’s *post-hoc* test was applied for multiple group comparisons. Statistical significance was defined as P< 0.05.

## 3 Results

### 3.1 Predicted targets for QSFZYL and LUAD

**Figure 2:**
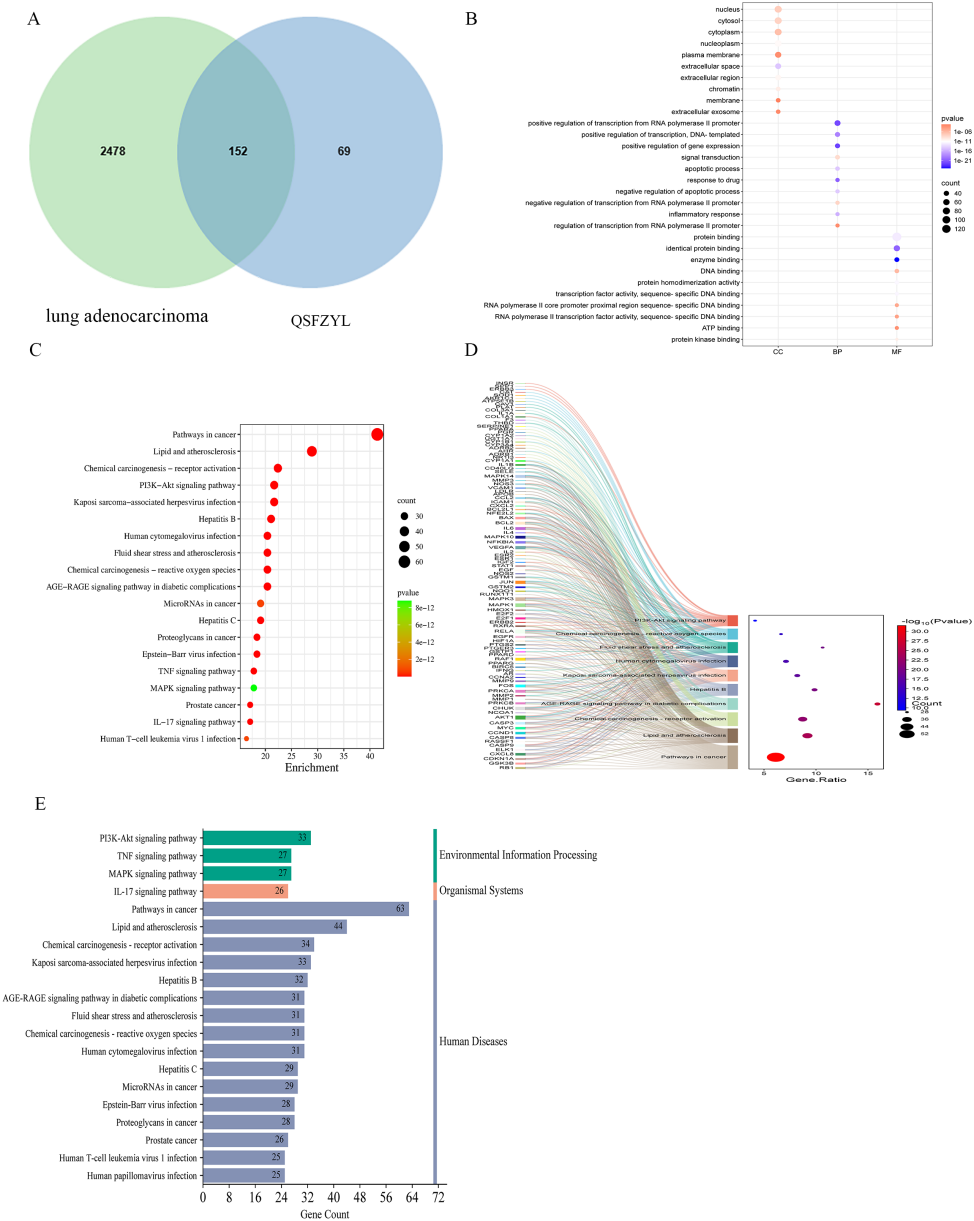
Network pharmacology results. **(A)** Venn diagram of qi–shen–fu–zheng–yi–liu treatment (QSFZYL) and lung adenocarcinoma intersection. **(B)** GO terms analysis include biological process, cellular component, and molecular function. **(C)** KEGG pathways were analyzed by the DAVID database. **(D)** The sankey diagram of the KEGG pathway analysis of the therapeutic targets of QSFZYL in lung adenocarcinoma treatment. The left rectangle nodes of the sankey diagram represent the therapeutic targets, the right rectangle nodes of the sankey diagram represent the KEGG pathways, and the lines represent the ownership of targets and pathways. **(E)** The KEGG type of the top 20 pathways based on KEGG enrichment analysis.

The TCMSP database was screened according to the specified criteria, yielding 157 active ingredients that corresponded to 2,914 targets. After converting these targets into gene names and further screening, a total of 221 unique targets were identified (**Supplementary Table S1**). Additionally, 2,214 targets were retrieved from the GeneCards database and 453 from the OMIM database, resulting in a total of 2,630 integrated targets. A Venn diagram was created using the Venny map website, revealing 152 intersecting genes (**Figure 2A**). The drug components and their predicted targets related to QSFZYL were imported into Cytoscape. The analysis results indicated that the most effective drug component of the fu–zheng tumor-suppressing formula was quercetin (MOL000098), followed by formononetin (MOL000392), kaempferol (MOL000422), lignocerotoxin (MOL000006), and isoflavones (MOL000417).

### 3.2 GO and KEGG enrichment analyses

**Figure 3:**
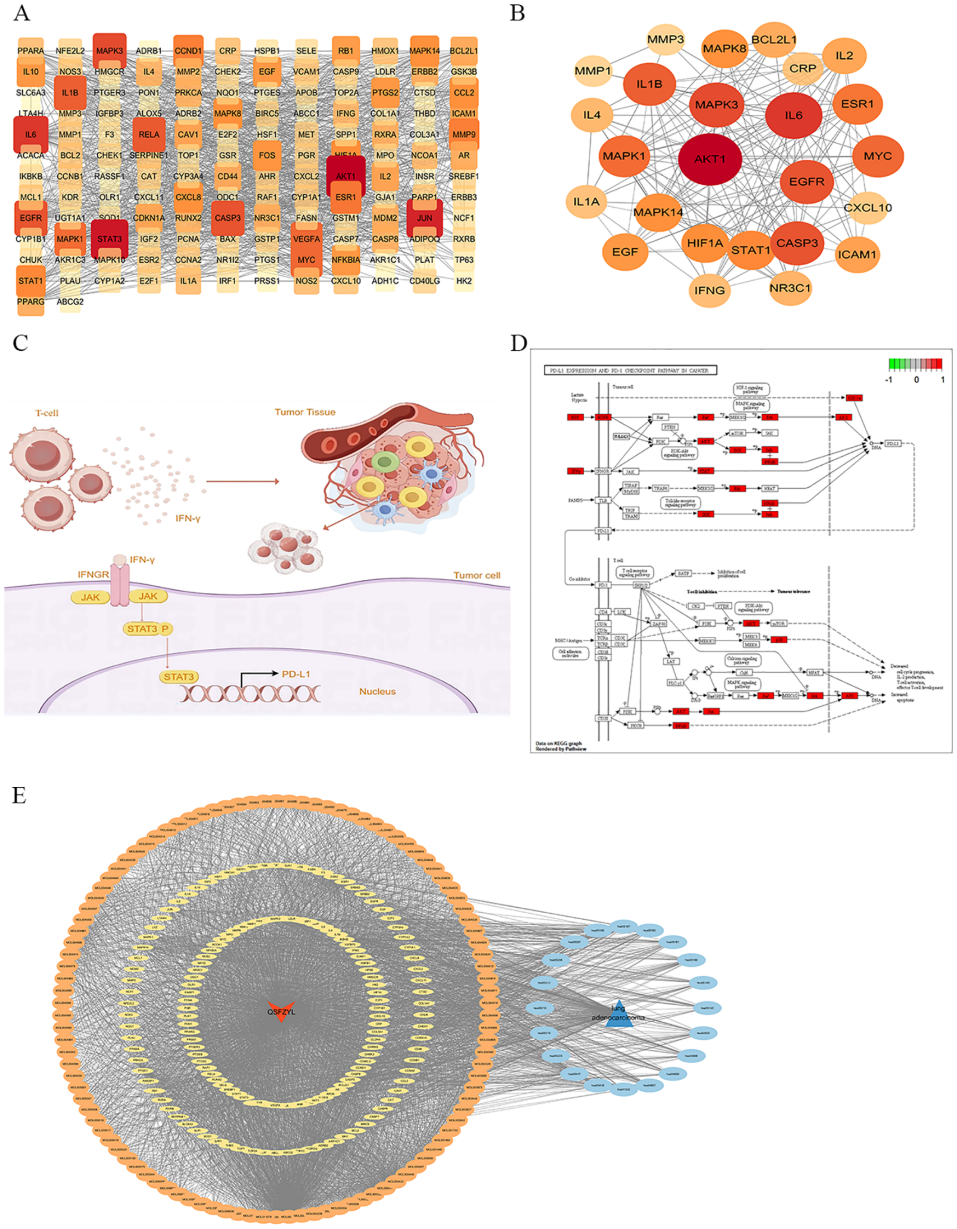
Network pharmacology results. **(A)** Protein–protein interaction (PPI) network demonstrating 152 protein targets plotted using the STRING database. **(B)** Protein–protein interaction (PPI) network demonstrating Top 25 protein targets plotted using the STRING database. **(C)** JAK/STAT signaling pathway. **(D)** PD-L1/PD-1 signaling pathway. **(E)** Drug disease active ingredient target pathway diagram, where red represents QSFZYL, blue represents lung adenocarcinoma, orange represents compounds, sky blue represents pathways, and yellow represents the target. Node size is proportional to the node degree.

To assess which of the lung adenocarcinoma-related biological process and signaling pathway may be affected by QSFZYL, GO and KEGG enrichments were performed by using the overlapping genes. As shown in **Figure 2B**, GO enrichment results were composed of three parts: cell component (CC, eg, nucleus and cytosol), biological process (BP, eg, positive regulation of transcription from RNA polymerase II promoter), and molecular function (MF, eg, protein binding). Moreover, the 20 top KEGG pathway enrichments were shown in the bubble chart (**Figure 2C**), which also suggested the possible mechanisms of QSFZYL resisting lung adenocarcinoma through multiple signaling pathways, lipid and atherosclerosis, chemical carcinogenesis-receptor activation, PI3K-Akt signaling pathway and etc (**Figure 2E**). The sankey diagram of the KEGG pathway analysis of the therapeutic targets of QSFZYL in lung adenocarcinoma treatment (**Figure 2D**). The above results suggest that the inhibition of lung adenocarcinoma by QSFZYL maybe associated with autophagy, indicating potential involvement of multiple biological processes, signaling pathways, or diseases. In addition, GO enrichment analysis revealed that the binding of STAT family proteins, positive regulation of tyrosine phosphorylation of STAT proteins, and cellular response to IFN-γ were associated with the treatment of lung adenocarcinoma using QSFZYL. KEGG enrichment analysis indicated associations with the JAK/STAT signaling pathways (**Figure 3C**) and PD-L1/PD-1 signaling pathways (**Figure 3D**).

### 3.3 Cross-gene protein mapping

After importing the 152 intersecting genes, the confidence interval was set to 0.700, and Cytoscape was used to construct the PPI network (**Figures 3A, B**). The five largest node targets identified were protein kinase 1 (AKT1), STAT3, JUN, interleukin-6 (IL-6), and mitogen-activated protein kinase 3 (MAPK3), which may be important targets for the treatment of lung adenocarcinoma with QSFZYL. To better understand the relationships among the drugs, compounds, targets, pathways, and diseases, a correlation network containing 280 nodes (106 compounds, 152 targets, and 20 pathways) and 3,387 edges was constructed (**Figure 3E**).

### 3.4 Molecular docking between core targets and key compound molecules

**Figure 4:**
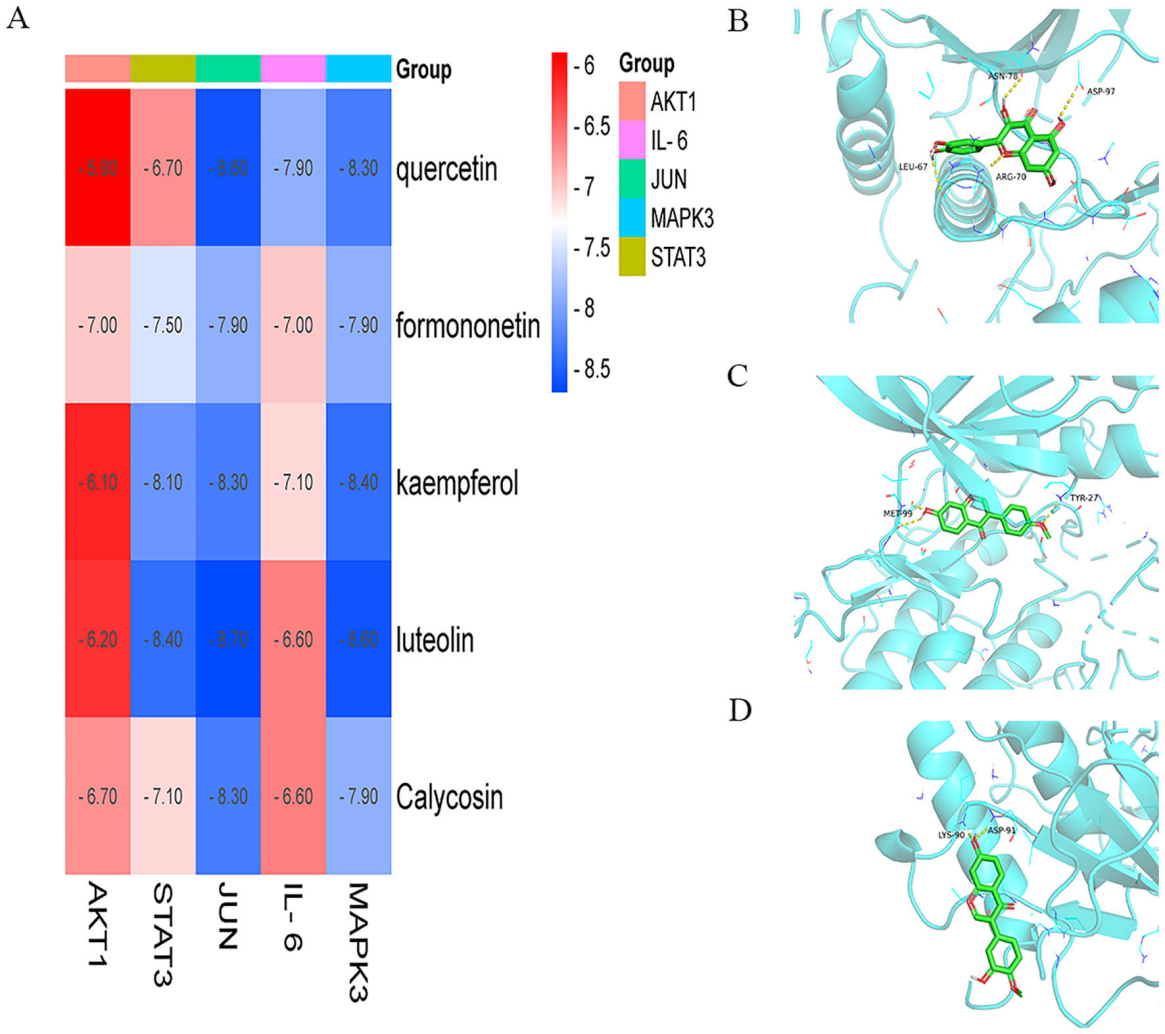
Molecular docking. **(A)** The docking score of critical ingredients of QSFZYL with hub target proteins. **(B)** Molecular docking protein of IL 6 and quercetin. **(C)** Molecular docking protein of STAT 3 and quercetin. **(D)** Molecular docking protein of STAT 3 and formononetin. **(E)** Molecular docking protein of STAT 3 and Calycosin.

To determine the binding pattern of the active compounds of the drug with the core targets, the top 5 active compounds of the drug and the top 5 core targets were selected from the PPI network and molecular docking was performed. The docking results show that the minimum binding efficiency of the active components of the drug with the core targets is lower than -5.0 Kcal/mol, indicating that they have good binding activity. The molecular docking results data were imported into imageGP (http://www.ehbio.com/), and a heat map was generated (**Figure 4A**). And some molecular docking diagrams are shown in **Figures 4B–D**. Red indicates a weaker binding, while blue indicates a stronger binding. The visual analysis of the processing results shows the interaction between the drug components and the core targets and the binding sites, and preliminarily verifies that QSFZYL mainly exerts its therapeutic effect on lung adenocarcinoma by acting on the corresponding core targets through the above active components.

### 3.5 Sample test chromatogram - mass spectrometry BPC plot

**Figure 5:**
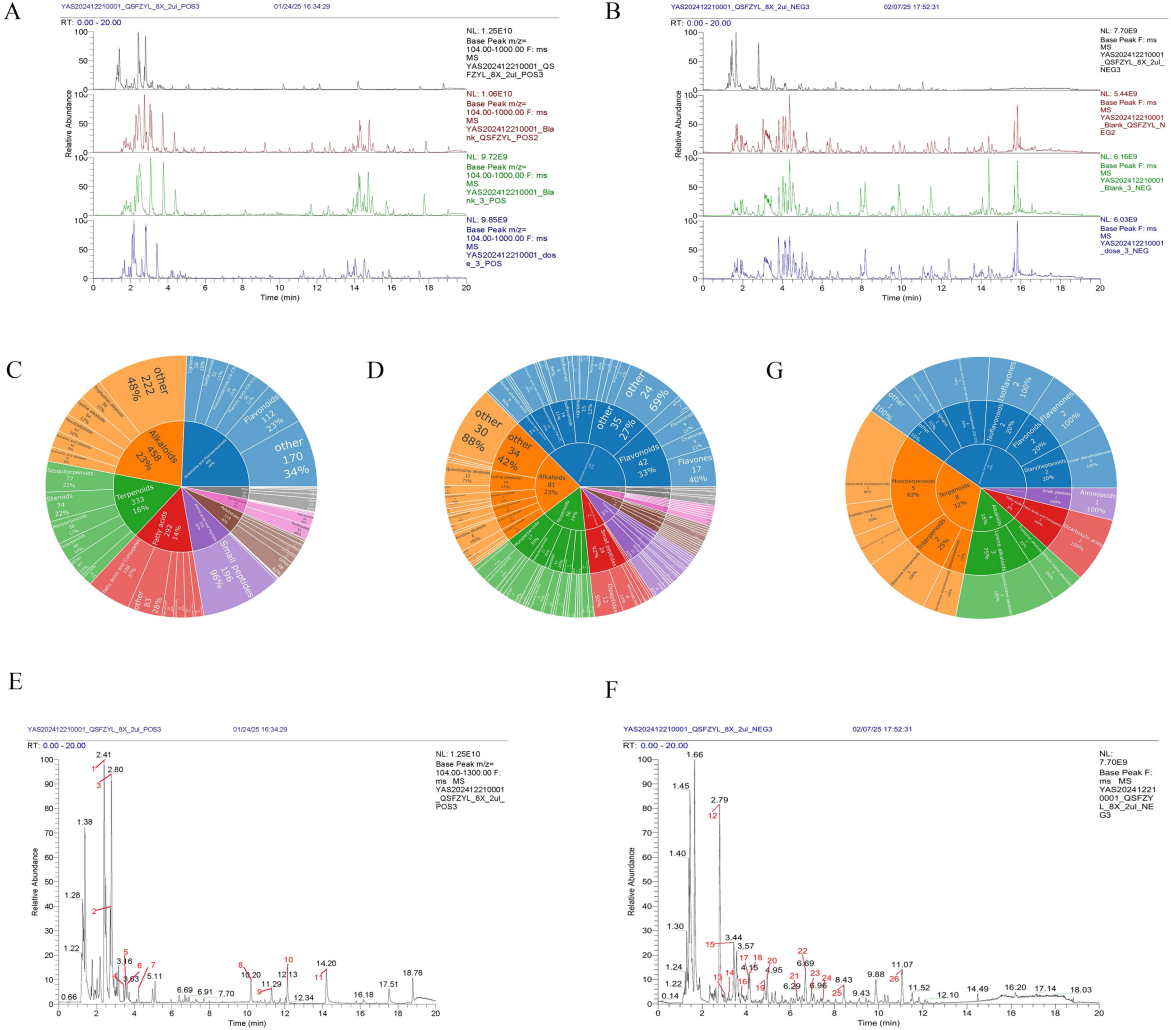
Chemical identification results. **(A)** BPC overlap profiles in positive ion mode for each group of samples. **(B)** BPC overlap profiles in negative ion mode for each group of samples. **(C)** Number of all identified compounds in each chemical classification. **(D)** Number of blood-entry compounds in each chemical classification. **(E)** BPC map-labelled peaks in QSFZYL positive ion mode. **(F)** QSFZYL BPC plot in negative ion mode - labelled peaks. **(G)** Number share of herbal labelled compounds in each chemical classification.

UHPLC-HRMS was used to analyze each group. Positive and negative ion base peak chromatograms (BPCs) were compared (**Figures 5A, B**). From the spectra, there is a certain difference in the spectra between the drug administration group and the blank control group.

### 3.6 Results of chemical identification

#### 3.6.1 Identification and classification of chemical compounds in the samples of traditional Chinese medicine and its drug delivery group

Compounds were identified via high-resolution mass spectrometry (HRMS) using a local TCM database (Zhongke New Life), with mass errors<25 ppm and fragment spectrum matching scores >0.7. The chemical compounds in the samples of traditional Chinese medicines and their administration groups were analyzed and identified, and the statistical results are shown in **Table 3**. The compounds identified in this experiment (compounds identified by combining positive and negative ions) were annotated according to the NPClassifier method, and the statistical results of the number of compounds identified in each pathway and major superclass are shown in **Table 4**, the proportion of each compound category is shown in **Figure 5C**, and the proportion of blood-entry compounds is shown in **Figure 5D**.

**Table 3:** Statistics on the number of compounds identified by positive and negative ion modes.

Detected Ion mode	Quantity of identified substance	Quantity of incoming substance
Positive ion mode (POS)	1402	193
Negative ion mode (NEG)	824	143
Total positive and negative ion modes	2032	336

Positive and negative ion modes combined: no double counting if the same compound is identified in both positive and negative ion modes.

**Table 4:** Compound type and quantity.

Pathway	SuperClass	Count
Alkaloids	Anthranilic acid alkaloids	37
Alkaloids	Lysine alkaloids	54
Alkaloids	Nicotinic acid alkaloids	42
Alkaloids	Pseudoalkaloids	47
Alkaloids	Tryptophan alkaloids	56
Alkaloids	other	222
Amino acids and Peptides	Oligopeptides	2
Amino acids and Peptides	Small peptides	196
Amino acids and Peptides	other	6
Amino acids and Peptides	β-lactams	1
Carbohydrates	Aminosugars and aminoglycosides	4
Carbohydrates	Nucleosides	35
Carbohydrates	Polyols	3
Carbohydrates	Saccharides	28
Carbohydrates	other	2
Fatty acids	Fatty Acids and Conjugates	108
Fatty acids	Fatty acyls	20
Fatty acids	Fatty amides	28
Fatty acids	Fatty esters	25
Fatty acids	Octadecanoids	28
Fatty acids	other	83
Polyketides	Aromatic polyketides	14
Polyketides	Cyclic polyketides	20
Polyketides	Macrolides	10
Polyketides	Phloroglucinols	5
Polyketides	Polycyclic aromatic polyketides	17
Polyketides	other	48
Shikimates and Phenylpropanoids	Flavonoids	112
Shikimates and Phenylpropanoids	Isoflavonoids	52
Shikimates and Phenylpropanoids	Lignans	50
Shikimates and Phenylpropanoids	Phenolic acids (C6-C1)	56
Shikimates and Phenylpropanoids	Phenylpropanoids (C6-C3)	55
Shikimates and Phenylpropanoids	other	170
Terpenoids	Diterpenoids	29
Terpenoids	Monoterpenoids	58
Terpenoids	Sesquiterpenoids	77
Terpenoids	Steroids	74
Terpenoids	Triterpenoids	48
Terpenoids	other	47
other	Meroterpenoids	11
other	Naphthalenes+Diterpenoids	2
other	Peptide alkaloids	3
other	Pseudoalkaloids	4

#### 3.6.2 Analysis of traditional Chinese medicines

In the base peak chromatograms (BPCs) of positive and negative ions for traditional Chinese medicines (TCM), peaks with higher abundance were identified by peak shape analysis and confirmed via secondary mass spectrometry. Chromatographic peaks were labeled sequentially (**Figures 5E, F**). A total of 26 peaks were annotated in this study (**Table 5**), with chemical classifications performed using NPClassifier (**Figure 5G**). By comparing the qualitative and quantitative results of the traditional Chinese medicine, the blank control group and the administration group, 12 of the 26 peaks labelled in the traditional Chinese medicine were blood-entry compounds (the intensity of the peaks in the administration group was more than three times higher than that of the peaks in the blank group). The representative compounds identified include astragalus Lysinealkaloids, Monoterpenoids, Isoflavonoids and Flavonoids.

**Table 5:** Results of chemical composition of BPC icon peaks.

No	m/z	RT/min	ppm	Adduct	Score	Compound_EN	SuperClass	Into blood or none
1	249.196	2.74	2.0	[M+H]+	0.9999	Matrine	Lysinealkaloids	Into_Blood
2	263.1752	3.07	1.4	[M+H]+	0.9993	Oxysophocarpine	Lysinealkaloids	Into_Blood
3	265.1908	3.13	0.1	[M+H]+	0.9994	Oxymatrine	Lysinealkaloids	Into_Blood
4	205.097	3.72	1.2	[M+H]+	0.997	L-Tryptophan	Smallpeptides	None
5	227.0911	3.84	0.8	[M+H]+	0.9065	Sarracenin	Monoterpenoids	None
6	247.144	3.92	0.2	[M+H]+	0.9388	Lenticine	Tryptophanalkaloids	Into_Blood
7	197.0809	4.53	0.5	[M+H-C6H10O5]+	0.9904	Sweroside	Monoterpenoids	None
8	269.0806	10.53	0.1	[M+H]+	0.9991	Formononetin	Isoflavonoids	Into_Blood
9	453.3361	11.61	0.3	[M+H-H2O]+	0.9889	18.beta.-Glycyrrhetinicacid	Triterpenoids	None
10	247.1327	12.43	0.9	[M+H]+	0.9669	Zederone	Sesquiterpenoids	None
11	149.0233	14.49	0.6	[M+H-C18H38O]+	0.9983	Di(2,6-dimethyl-4-heptyl)phthalate	NA	None
12	255.0512	3.03	0.1	[M-H]-	0.7555	(2R,3S)-Piscidicacid	NA	Into_Blood
13	353.0881	3.29	3.8	[M-H]-	0.9802	Caffeoylquinicacid	Phenylpropanoids(C6-C3)	Into_Blood
14	375.1302	3.45	0.9	[M-H]-	0.9475	Loganicacid	Monoterpenoids	Into_Blood
15	451.1464	3.69	2.0	[M+HCO2]-	0.9592	Morroniside	Monoterpenoids	Into_Blood
16	175.0607	4.04	2.3	[M-H]-	0.9744	2-Isopropylmalicacid	FattyAcidsandConjugates	None
17	403.1249	4.37	1.5	[M-H]-	0.8533	Secoxyloganin	Monoterpenoids	Into_Blood
18	179.0345	4.41	2.6	[M-H]-	0.9992	Grevillicacid	Phenylpropanoids(C6-C3)	None
19	549.162	5.05	2.4	[M-H]-	0.9886	Liguiritigenin-7-O-beta-D-apiosyl-4’-O-beta-D-glucosi	Flavonoids	None
20	417.1196	5.22	2.2	[M-H]-	0.9789	Liquiritin	Flavonoids	Into_Blood
21	187.0974	6.55	1.5	[M-H]-	0.9982	Azelaicacid	FattyAcidsandConjugates	None
22	373.1296	6.87	1.2	[M-H]-	0.9266	Wikstromol	Lignans	None
23	315.1606	7.22	2.0	[M-H]-	0.9942	Hannokinol	Diarylheptanoids	Into_Blood
24	283.0616	7.7	3.5	[M-H]-	0.9943	Calycosin	Isoflavonoids	None
25	373.1659	8.69	0.0	[M-H]-	0.9579	1,7-Bis(3,4- dihydroxyphenyl)heptan-3-yl acetate	Diarylheptanoids	None
26	821.3982	11.31	3.8	[M-H]-	0.9359	(2S,3S,4S,5R,6R)-6-[(2R,3R,4S,5S,6S)-2-[[(3S,6aR,6bS,8aS,12aR,14bS)-11-carboxy-4,4,6a,6b,8a,11,14b-heptamethyl-14-oxo-2,3,4a,5,6,7,8,9,10,12,12a,14a-dodecahydro-1H-picen-3-yl]oxy]-6-carboxy-4,5-dihydroxyoxan-3-yl]oxy-3,4,5-trihydroxyoxane-2-carboxylicacid	Triterpenoids	None

(Header: NO: Serial number; m/z: parent ion mass-to-charge ratio; RT/min: retention time/min; ppm: first-degree mass deviation; Adduct: adduct; Score: second-degree mass spectrometry matching score; Compound EN: English name of compound; Compound CN: Chinese name of compound; SuperClass: Classification of compound; Into Blood or None: Compound in blood or not in blood; NA: Not available; *Identification of compound as control).

### 3.7 Primary culture, isolation, and identification of BMSCs

Primary BMSCs began to adhere to culture plates 24 hours post-extraction, displaying diverse morphologies, predominantly shuttle shape. Flow cytometry revealed high expression of CD44 and CD105, while CD34 and CD45 were absent, confirming their identity as bone marrow mesenchymal stem cells (BMSCs).

### 3.8 Detection of IFN-γ overexpression in BMSCs cells by Western blotting

**Figure 6:**
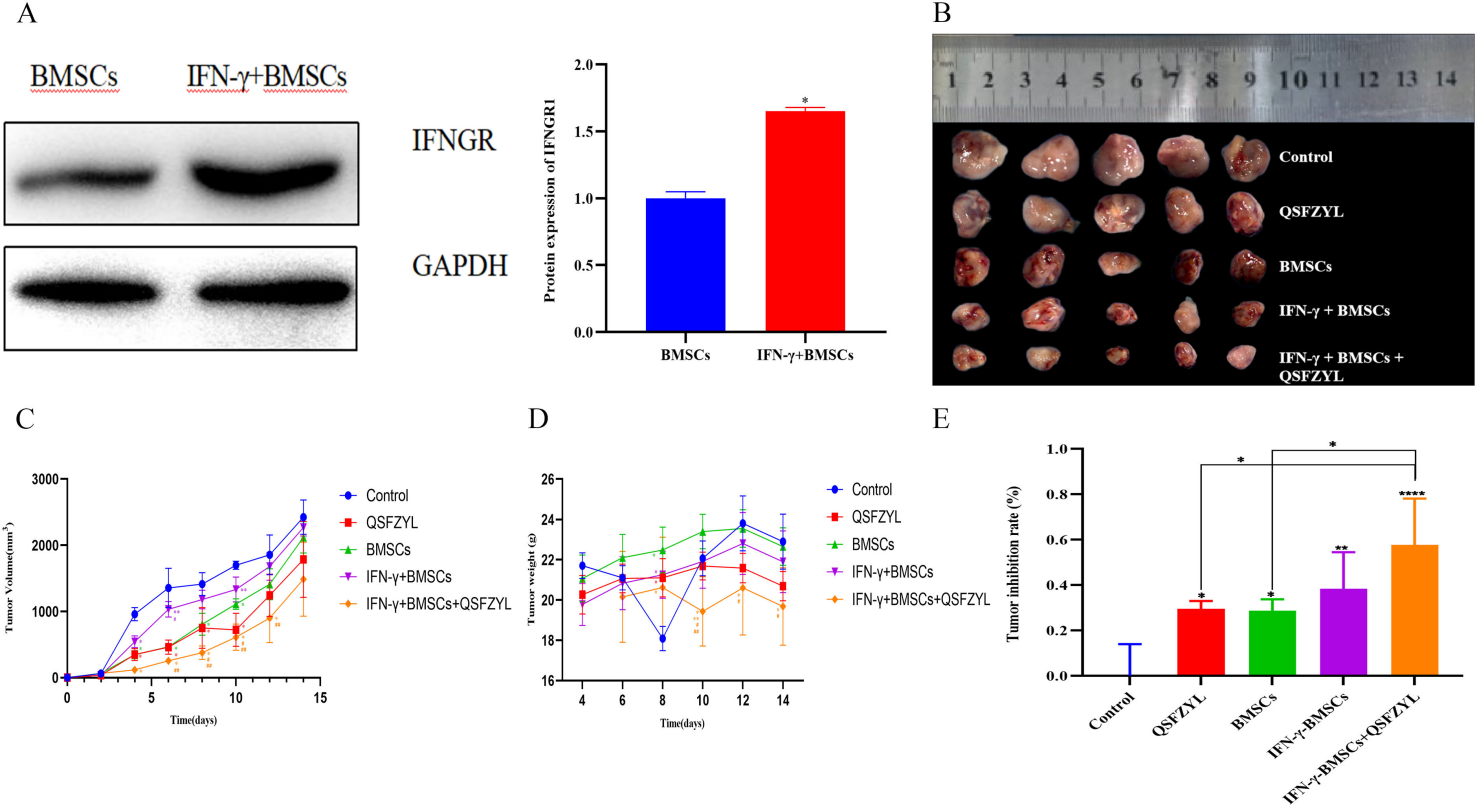
Tumor volume changes in tumor-bearing mice. **(A)** Transfection efficiency of IFN-γ (*P< 0.05, significantly different from the BMSC group). **(B)** Comparison of animal tumors. **(C)** Tumor growth curve for tumor-bearing mice in each group (*P< 0.01 vs. control; **P< 0.01 vs. QSFZYL; #P< 0.01 vs. BMSCs; ##P< 0.01 vs. IFN-γ + BMSCs). **(D)** Weight of tumor-bearing mice in each group (*P< 0.01 vs. control; **P< 0.01 vs. QSFZYL; #P< 0.01 vs. BMSCs; ##P< 0.01 vs. IFN-γ + BMSCs). **(E)** Tumor inhibition rate in each group (*P< 0.05; **P< 0.01; ****P< 0.0001).

To validate IFN-γ transfection efficiency, IFNGR1 expression was analyzed in the IFN-γ + BMSC and BMSC groups via Western blotting (**Figure 6A**). The results demonstrated a significant upregulation of IFNGR1 in the IFN-γ + BMSC group compared to controls (P< 0.05), confirming successful IFN-γ transfection.

### 3.9 Tumor volume changes in tumor-bearing mice

To investigate the effects of QSFZYL combined with IFN-γ on anti-lung cancer *in vivo*, we constructed a mouse lung adenocarcinoma model. After successful modelling, the tumor volume and body weight of the mice were measured every other day. The change in tumor volume for each group was plotted as a growth curve. As shown in **Figure 6B**, tumor volumes in the BMSCs, QSFZYL, IFN-γ + BMSCs + QSFZYL, and IFN-γ + BMSCs groups were significantly smaller than the control group on day 8 post-treatment (P< 0.05). Notably, the IFN-γ + BMSCs + QSFZYL group exhibited superior tumor suppression compared to the IFN-γ + BMSCs group on days 8, 10, and 12 (P< 0.05) and to the BMSCs group on days 8 and 10 (P< 0.05). Weight changes (**Figure 6C**) and tumor growth inhibition rates (**Figures 6D, E**) further validated these trends, with the IFN-γ + BMSC + QSFZYL group showing significant reductions in body weight and enhanced inhibition rates compared to other groups (P< 0.05).

### 3.10 Tumor apoptosis in tumor tissue of mice

**Figure 7:**
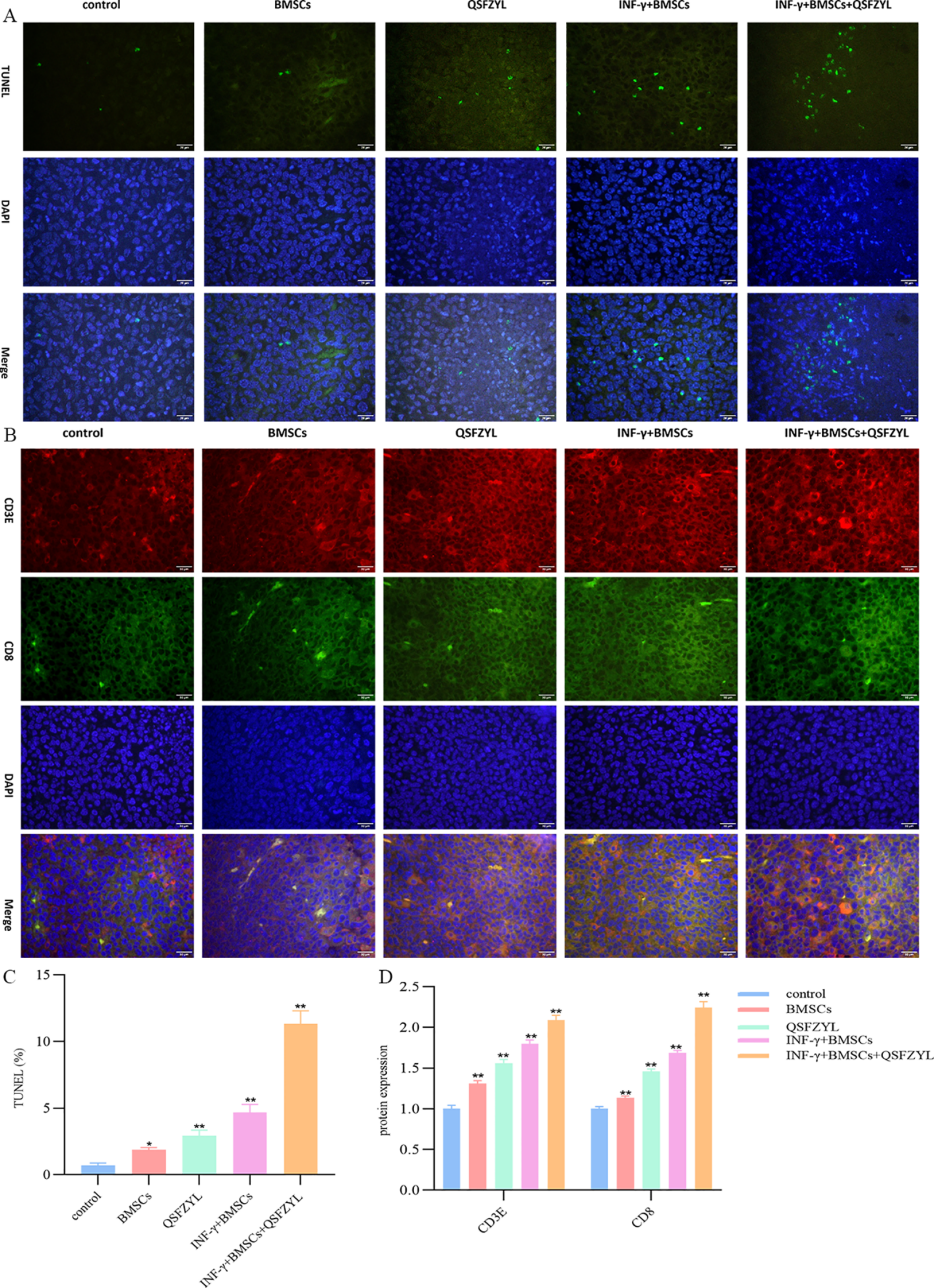
QSFZYL exerted anti-tumor effects through enhancing the immune response in Lewis-bearing mice. **(A)** Immunofluorescence of the CD3E (red) and CD8 (green) combined with DAPI staining (blue) in tumor tissue from Lewis-bearing mice. **(B)** ImageJ analysis of tumor cell CD3E and CD8 levels in each group. *p<0.05 and **p<0.01vs Model group **(C)** TUNEL staining marking primary apoptotic tumor cells; **(D)** ImageJ analysis of tumor cell apoptosis levels in each group. * P<0.05 and **p<0.01vs Model group.

TUNEL staining, which marks apoptotic tumor cells, revealed large areas of apoptosis in the BMSC, QSFZYL, IFN-γ + BMSC, and IFN-γ + BMSC + QSFZYL group intervention group (**Figures 7A, C**), with a significantly higher apoptotic area compared to the model group (P< 0.01).

### 3.11 QSFZYL enhanced the immune response in LUAD

The immunostaining results in **Figures 7B, D** showed that, compared to the model group, the expression of CD3 and CD8 was significantly higher in the BMSC, QSFZYL, IFN-γ + BMSC, and IFN-γ + BMSC + QSFZYL group (P< 0.01, P< 0.001). These results indicated that IFN-γ + BMSC + QSFZYL could activate the immune response in LUAD.

### 3.12 JAK2, STAT3, and PD-L1 protein expression in LUAD tissues

**Figure 8:**
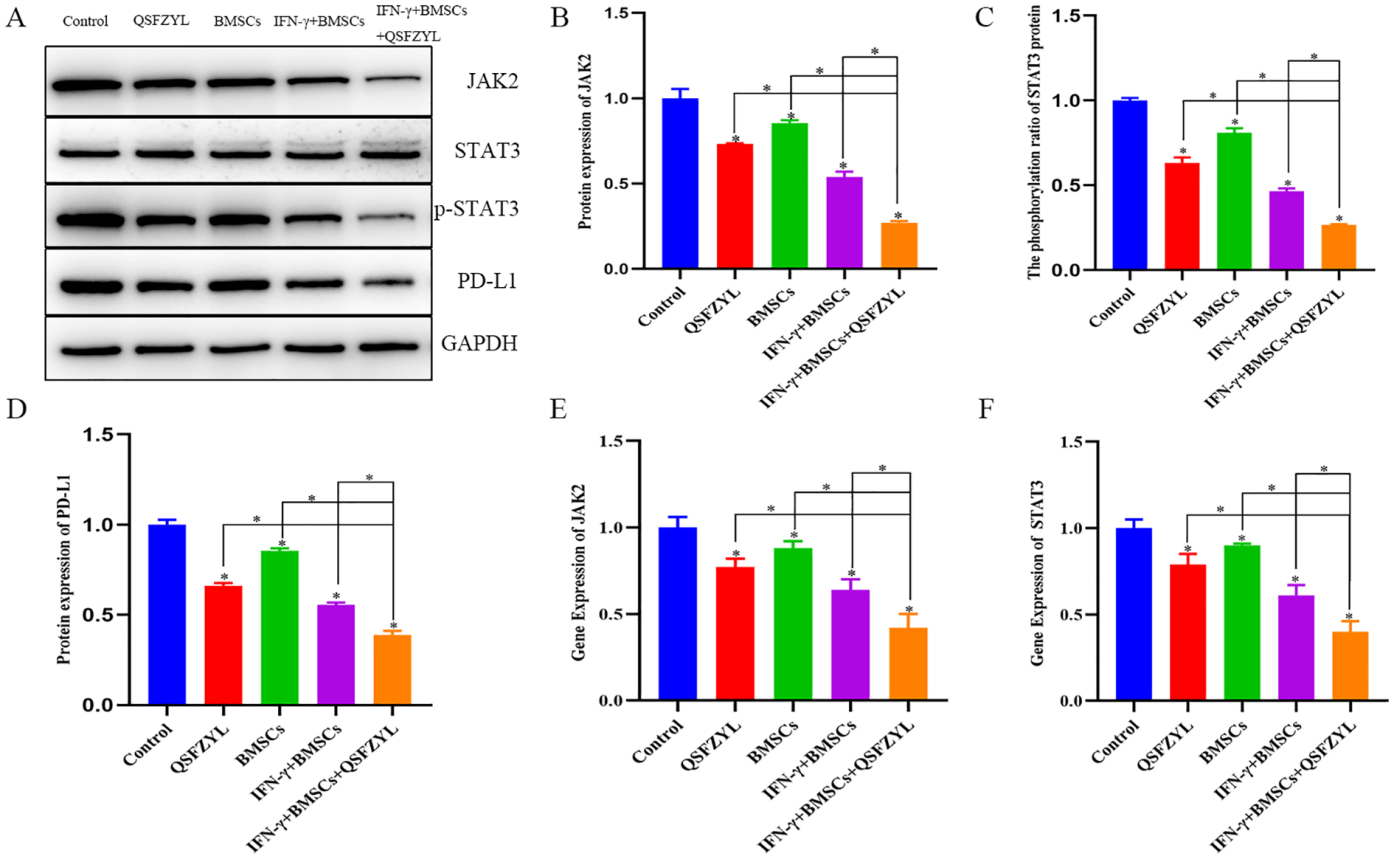
Protein expression and gene expression in lung adenocarcinoma tissues. **(A–D)** Expression of PD-L1, JAK2, and p-STAT3/STAT3 proteins in tumor tissues. **(E, F)** JAK2 and STAT3 gene expression in tumor tissues. Data are means ± standard deviation (SD) of three independent experiments. Asterisks indicate significant differences (*P< 0.05).

To investigate signaling events mediated by QSFZYL in combination with IFN-γ-modified BMSCs, we examined the expression levels of JAK2, STAT3, and PD-L1 (**Figures 8A-D**). Compared to the control group, JAK2, STAT3, and PD-L1 protein expression was reduced in lung cancer tissues of mice from the BMSCs, QSFZYL, IFN-γ + BMSCs, and IFN-γ + BMSCs + QSFZYL groups. Notably, JAK2, STAT3, and PD-L1 protein expression was significantly lower in lung cancer tissues of mice in the IFN-γ + BMSCs + QSFZYL group compared to the BMSCs, QSFZYL, and IFN-γ + BMSCs groups (P< 0.05).

### 3.13 JAK2 and STAT3 gene expression in LUAD tissues

To further investigate the effects of QSFZYL combined with IFN-γ on the JAK2/STAT3 signaling pathway in lung adenocarcinoma, we performed qPCR analysis for JAK2 and STAT3 in tumor tissues of mice from each intervention group (**Figures 8E, F**). The results showed significantly decreased JAK2 and STAT3 mRNA levels in the BMSC, QSFZYL, IFN-γ + BMSC, and IFN-γ + BMSC + QSFZYL groups compared to controls (P< 0.05). The IFN-γ + BMSC + QSFZYL group exhibited the lowest expression levels, differing significantly from all other groups (P< 0.05).

## 4 Discussion

TCM can complement radiotherapy and chemotherapy by addressing drug resistance, enhancing clinical symptom management, and mitigating the side effects of radiotherapy through multi-target and multi-pathway synergistic effects (19). The QSFZYL treatment is based on a traditional treatment (fu–zheng–yi–liu) developed by Zhao Jianxiong, a TCM practitioner in Gansu Province, China. It was modified with the addition of Sophora flavescens Aiton and Glycyrrhiza uralensis Fisch. ex DC. to enhance its synergistic effects of reduced toxicity and increased efficacy. Matrine (Mat), a component extracted from Sophora flavescens Ait, has a wide spectrum of pharmacological effects. Glycyrrhizin (Gly), a major active constituent of licorice (Glycyrrhiza glabra) root, has various pharmacological effects. Studies have shown that matrine and glycyrrhizin produce anticancer effects *in vivo* and *in vitro*, respectively (20, 21). Both have the ability to protect the liver in cases of acute liver injury caused by various chemicals. Serological evidence (22) suggested that matrine and glycyrrhizin could inhibit the precancerous stage of tumors and had a positive effect on tumor prevention. Experimental studies in mice showed that the combination of matrine and glycyrrhizin significantly reduced the hepatotoxicity of acetaminophen in mice. Moreover, the combination of matrine and glycyrrhizic acid increased the content of CD4+ and CD8+ immune cells in the peripheral blood of mice, indicating that their combination has the effect of regulating cellular immunity. Studies have shown that the combination of matrine and glycyrrhizic acid can reduce the absorption of glycyrrhizic acid and accelerate its decomposition, thereby reducing the accumulation of glycyrrhizic acid in the body and reducing side effects such as sodium and water retention, hypertension, and hypokalemia caused by glycyrrhizic acid (23). At the same time, studies have shown that the combined application has nonspecific anti-inflammatory effects (24). Studies have found that glycyrrhizic acid can slow down the metabolism of matrine and reduce its toxic metabolites by inhibiting CYP450 enzymes (such as CYP3A4) (25). Glycyrrhizic acid flavonoids have an antagonistic effect on the oxidative stress of hepatocytes induced by matrine. In addition, glycyrrhizic acid forms complexes with alkaloids (such as matrine), which can significantly enhance the induced apoptosis of hepatoma cells (HepG2), reduce the concentration of free toxic components, and enhance antioxidant capacity by up-regulating the Nrf2 pathway. In the Qishen Yiqi Formula, licorice can significantly enhance the anti-myocardial fibrosis effect of Sophora flavescens and simultaneously alleviate liver damage. In this study, to explore the molecular mechanisms of QSFZYL in the treatment of LUAD, we applied a network pharmacology approach to predict key targets and potential pathways. A total of 152 intersecting genes were identified between the active ingredients of QSFZYL and lung adenocarcinoma-related genes. Through PPI mapping, five key targets were highlighted: AKT1, STAT3, JUN, IL-6, and MAPK3. The active compounds of QSFZYL included quercetin, formononetin, kaempferol, lignocerotoxin, and isoflavones. GO analysis revealed that the effects of QSFZYL on lung adenocarcinoma are associated with the binding of STAT family proteins, positive regulation of tyrosine phosphorylation, and modulation of STAT proteins. Cellular responses to IFN-γ were also implicated. KEGG pathway analysis suggested that the QSFZYL mechanism of action may involve the JAK/STAT signaling pathway.

UHPLC-HRMS analysis identified 26 compounds in QSFZYL, of which 12 were compounds that entered the blood. The representative compounds identified include Lysinealkaloids (Matrine, Oxysophocarpine, Oxymatrine); Monoterpenoids (Sarracenin Sweroside, Loganicacid Morroniside, Secoxyloganin); Isoflavonoids (Formononetin, Calycosin) and Flavonoids (Liquiritin). Matrine exhibits a variety of pharmacological properties, including anti-inflammatory, antitumor, and antifibrotic effects (26), the mechanisms underlying its antitumor activity are complex, including cell proliferation, migration, and apoptosis (27, 28). In the past few years, there has been increasing evidence that Picrasidine Oxidized Picrasines may have some promising therapeutic potential in cancer, such as their effects on a number of cancer cell lines, including those derived from gastric cancer, cervical cancer, leukemia, hepatocellular carcinoma, breast, pancreatic, and lung cancers (29). Oxysophocarpine is one of the major bioactive alkaloids extracted from Ginseng Ait, S alopecuroides L, and other legumes of Sophora japonica. In terms of antitumor functions, Oxysophocarpine has been determined to have significant inhibitory effects on oral squamous cell carcinoma and hepatocellular carcinoma (30). Oxymatrine possesses a variety of pharmacological effects, including antiviral, antifibrotic and other immunomodulatory effects, effectively inhibits CAF activation and promotes TIL infiltration in tumors, and has now been shown to inhibit tumor growth in a variety of tumor cell lines, including hepatocellular carcinomas, lung carcinomas, colorectal carcinomas, and breast carcinomas, as well as to suppress tumor growth through the regulation of miRNAs and a variety of signaling pathways, including STAT5, EGFR, NF- κB and PI3K/Akt in tumor development, progression and drug resistance (31–33). Monoterpenes, a subclass of terpenoids, are common secondary metabolites found in the essential oils of aromatic plants. Their antitumor properties, including antiproliferative, apoptotic, antiangiogenic and antimetastatic effects, and other biological activities (34). Isoflavones have been found to induce apoptosis, interfere with proliferative molecular pathways, and even reduce tumor angiogenesis. In addition, population-level studies have emerged that correlate consumption of isoflavonoids with a reduced risk of lung cancer (35). Formononetin plays an important role in inhibiting the ability of cancer cells to proliferate, invade, and metastasize by targeting major signaling pathways at the junction of interconnected pathways. It also induces apoptosis and cell cycle arrest by regulating mediator proteins. In addition, formononetin regulates the tumor microenvironment by inactivating the ERK1/2 pathway and nuclear fibrillar protein A/C signaling, and has been reported to inactivate the JAK/STAT, PKB or AKT, and mitogen-activated protein kinase pathways, and to inhibit cell migration, invasion, and angiogenesis in human carcinoma cells (36). Flavonoids can regulate the activity species of enzymes involved in the clearance of reactive oxygen species, induce cell cycle arrest, apoptosis and autophagy, and inhibit the proliferation and invasiveness of cancer cells through different pathways in terms of their anti-cancer effects (37, 38).

Research utilizing bone marrow mesenchymal stem cells (BMSCs) as therapeutic tumor carriers has gained significant traction due to their unique tumor-targeting capabilities (39). BMSCs have been used to carry a variety of anticancer drugs and have exhibited notable inhibitory effects in tumor animal models (40). For example, BMSCs transfected with the IL-12 gene demonstrated strong targeting of tumor tissues, further validating their reliability as tumor biotherapeutic carriers (41). Although BMSCs share some self-renewal potential with tumor cells (42, 43),. However, significant differences in the expression of proliferative genes between BMSCs and cancer cells have been reported. BMSCs express a number of cell surface markers, including CD44 and CD105. CD44 has been recognized as a marker for CSCs and a therapeutic target for a variety of cancers.CD44 is involved in several immune response processes. Upon activation through the T cell receptor (TCR), CD44 expression is upregulated on naïve T cells. In cancer, CD44 is highly expressed in gastric cancer and is associated with gastric immune invasion.CD44 can be used as a prognostic biomarker in gastric cancer (44). In triple-negative breast cancer (TNBC) and non-small cell lung cancer (NSCLC), CD44 positively regulates the expression of PD-L1 by binding to the regulatory region of the PD-L1 locus (45).CD105 is highly expressed in tumor cells and vascular endothelial cells around or inside tumors (including HCC), and it is an important marker for neovascularization in tumors. In recent years, the targeting of CD105 anti-tumor therapy has become a research hotspot (46). It is now shown that CD105 promotes invasion and metastasis of hepatocellular carcinoma cells by increasing VEGF expression (47). In one study, CD44-/CD105- cells were injected subcutaneously into NOD SCID mice and tumor growth was monitored by MRI and PET/CT. The results showed that CD44-/CD105- cells not only induced tumor growth in mice, but also that the tumors had a longer T1 time and a different metabolic pattern than other tumors (48).

IFN-γ is a therapeutic agent with multiple immunomodulatory, antiviral, antimicrobial, antitumor, and proinflammatory activities; it is primarily involved in host defense against infections and tumor surveillance, and plays a critical role in inhibiting tumor development and progression by stimulating the immune system (49). IFN-γ also promotes apoptosis, inhibits cell proliferation, and prevents tumor angiogenesis (50). Its strong inhibitory effects have been demonstrated using various tumor models, including bladder cancer, colorectal cancer, ovarian cancer, adult T-cell leukemia, human pancreatic cancer cells, and non-small cell lung cancer (NSCLC), highlighting its broad potential in healthcare (51). In this study, the IFN-γ gene was transfected into BMSCs to explore the key role of IFN-γ combined with QSFZYL in treating LUAD. In tumor-bearing mice, QSFZYL, IFN-γ-BMSCs, and IFN-γ-BMSCs-QSFZYL significantly reduced tumor volume versus controls. Notably, the IFN-γ-BMSCs-QSFZYL outperformed single-agent therapies, demonstrating enhanced efficacy over QSFZYL alone or BMSCs transduced with IFN-γ. Importantly, QSFZYL combined with IFN-γ-BMSCs surpassed outcomes from the QSFZYL formula alone, underscoring TCM’s potential to reduce toxicity and amplify targeted BMSC therapies.

The tumor microenvironment (TME) comprises an extracellular matrix, mesenchymal cells, fibroblasts, and tumor-infiltrating lymphocytes (TILs) (52). The balance between pro- and antitumor factors in the TME critically determines tumor progression and metastasis (53). Infiltration of CD3^+^, CD4^+^, and CD8^+^ T lymphocytes correlates positively with prognosis in laryngeal squamous cell carcinoma (LSCC) (54). Some studies have also found that anti-angiogenic agents play a key role by normalizing blood vessels and acting on immune cells, with the combination group showing a similarly significant increase in the proportion of CD3, CD4, and CD8 T cells compared to the other groups. In NSCLC, high densities of tumor-infiltrating T-lymphocytes and B-cells associate with prolonged survival (55). A study analyzing 680 NSCLC resection specimens revealed that tertiary lymphoid structures (TLS) correlate with plasma cell (CD138^+^) and lymphocyte (CD3^+^, CD8^+^, FOXP3^+^) infiltration, while higher tumor mutational burden predicts increased peripheral TLS (56). In our prior work, we stably expressed IFN-γ in bone marrow mesenchymal stem cells (BMSCs) via lentiviral transfection and established a subcutaneous lung adenocarcinoma model in C57BL/6J mice. IFN-γ-overexpressing BMSCs significantly reduced Treg levels, inhibited tumor immune evasion, and enhanced anti-tumor immunity in mice (6). The immunostaining results in **Figure 3A** showed that, compared to the model group, the expression of CD3 and CD8 was significantly higher in the BMSC, QSFZYL, IFN-γ + BMSC, and IFN-γ + BMSC + QSFZYL group, consistent with this conclusion. Our study also confirmed that BMSCs overexpressing IFN-γ combine with QSFZYL significantly increased the area of apoptosis-positive regions and in mouse tumors, further confirming the effectiveness of their antitumor actions. Previous experiments revealed that IFN-γ-overexpressing BMSCs upregulated pro-apoptotic BAX expression, downregulated anti-apoptotic BCL2, and promoted apoptosis in cancer cells. Furthermore, these cells attenuated inflammatory injury in the lung tissues of tumor-bearing mice, reduced regulatory T cell (Treg) levels to inhibit tumor immune evasion, and decreased the expression of PI3K/AKT and PD-L1.

The JAK/STAT signaling pathway plays a crucial role in cancer development and is particularly important in lung cancer research (57, 58). In lung cancer, JAK2 expression and PD-L1 expression have been shown to be correlated (59). Additionally, aberrant expression of STAT3 has been linked to lung carcinogenesis, and STAT3 inhibitors have been shown to inhibit the JAK/STAT3 signaling pathway *in vitro* (60, 61). When IFNs bind to their respective receptors, receptor-associated JAK is phosphorylated and activated, which subsequently phosphorylates and activates various members of the STAT family. In this study, we investigated whether IFN-γ-transfected BMSCs combined with QSFZYL affects the progression of lung adenocarcinoma through the JAK2/STAT3 pathway through Western blotting and qPCR analyses of the expression of JAK2/STAT3 pathway-related proteins and genes in intervention groups. The Western blotting results revealed that, compared with the control group, the BMSC, QSFZYL, IFN-γ + BMSC + QSFZYL, and IFN-γ-BMSCs groups exhibited reduced PD-L1, JAK2, STAT3, and p-STAT3 expression in lung cancer tissues. Furthermore, PD-L1, JAK2, and p-STAT3/STAT3 expression levels were significantly lower in the IFN-γ + BMSC + QSFZYL group than in the BMSC, QSFZYL, and IFN-γ-BMSCs groups. The qRT-PCR results corroborated these findings, showing lower PD-L1, JAK2, and p-STAT3/STAT3 expression levels of in the BMSC, QSFZYL, IFN-γ + BMSC + QSFZYL, and IFN-γ + BMSC groups than in the control group. These results further support the hypothesis that IFN-γ-transfected BMSCs combined with QSFZYL may inhibit lung adenocarcinoma proliferation by suppressing the JAK2/STAT3 pathway.

There are some limitations of this study. Although network pharmacology and *in vivo* experiments confirmed JAK/STAT as a signaling pathway for LC treatment by QSFZQL, relevant *in vitro* experimental validation is still lacking, and the network pharmacology revealed that QSFZQL targeting AKT1, STAT3, JUN, IL-6 and MAPK3 intervened to inhibit the proliferation of lung adenocarcinoma needs to be confirmed by further experiments. In addition, the efficacy-toxicity network of component-target-pathogenic gene we constructed ignores the path loss that may occur during gene transmission. In future studies, we hope to further improve the model and try to eliminate the effect of path loss in the gene transfer process on the final result.

## 5 Conclusion

In conclusion, this study demonstrates that QSFZYL combined with IFN-γ-overexpressing BMSCs exerts significant inhibitory effects on LUAD growth in mouse models. These effects are mediated through multiple synergistic mechanisms, including suppression of tumor cell proliferation, induction of apoptosis, promoted infiltration of CD3 and CD8 T cell, inhibition of the JAK2/STAT3 signaling pathway, and downregulation of PD-L1 expression. Notably, compared to single-agent therapies, IFN-γ-BMSCs+QSFZYL exhibited enhanced therapeutic efficacy. However, the precise molecular mechanisms underlying the anti-tumor effects of QSFZYL in conjunction with IFN-γ-BMSCs require further elucidation, particularly regarding its pharmacological interactions and immunomodulatory pathways. This work provides a foundational framework for understanding the role of TCM in cancer therapy and highlights its potential to synergize with conventional treatments.

## Data Availability

The original contributions presented in the study are included in the article/**Supplementary Material**. Further inquiries can be directed to the corresponding author.
