# Supplementary material for: Network pharmacology and UHPLC-HRMS reveal the mechanism of QSFZYL and BMSCs overexpressing IFN-γ against lung adenocarcinoma
Source: Front Immunol. 2025 Jun 26;16:1593121. doi: 10.3389/fimmu.2025.1593121 (PMC12240740; doi:10.3389/fimmu.2025.1593121)
Supplement: Supplementary file 1 [file Table1.docx]

Supplementary Table 1 Active ingredients of QSFZYL

| Latin scientiﬁc name | Chinese name | Mol ID | Molecule Name | OB (%) | DL |
| --- | --- | --- | --- | --- | --- |
| Glycyrrhiza uralensis Fisch | gancao | MOL001484 | [Inermine](https://old.tcmsp-e.com/molecule.php?qn=1484) | 75.18 | 0.54 |
| Glycyrrhiza uralensis Fisch | gancao | MOL001792 | [DFV](https://old.tcmsp-e.com/molecule.php?qn=1792) | 32.76 | 0.18 |
| Glycyrrhiza uralensis Fisch | gancao | MOL000211 | [Mairin](https://old.tcmsp-e.com/molecule.php?qn=211) | 55.38 | 0.78 |
| Glycyrrhiza uralensis Fisch | gancao | MOL002311 | [Glycyrol](https://old.tcmsp-e.com/molecule.php?qn=2311) | 90.78 | 0.67 |
| Glycyrrhiza uralensis Fisch | gancao | MOL000239 | [Jaranol](https://old.tcmsp-e.com/molecule.php?qn=239) | 50.83 | 0.29 |
| Glycyrrhiza uralensis Fisch | gancao | MOL002565 | [Medicarpin](https://old.tcmsp-e.com/molecule.php?qn=2565) | 49.22 | 0.34 |
| Glycyrrhiza uralensis Fisch | gancao | MOL000354 | [isorhamnetin](https://old.tcmsp-e.com/molecule.php?qn=354) | 49.6 | 0.31 |
| Glycyrrhiza uralensis Fisch | gancao | MOL000359 | [sitosterol](https://old.tcmsp-e.com/molecule.php?qn=359) | 36.91 | 0.75 |
| Glycyrrhiza uralensis Fisch | gancao | MOL003656 | [Lupiwighteone](https://old.tcmsp-e.com/molecule.php?qn=3656) | 51.64 | 0.37 |
| Glycyrrhiza uralensis Fisch | gancao | MOL003896 | [7-Methoxy-2-methyl isoflavone](https://old.tcmsp-e.com/molecule.php?qn=3896) | 42.56 | 0.2 |
| Glycyrrhiza uralensis Fisch | gancao | MOL000392 | [formononetin](https://old.tcmsp-e.com/molecule.php?qn=392) | 69.67 | 0.21 |
| Glycyrrhiza uralensis Fisch | gancao | MOL000417 | [Calycosin](https://old.tcmsp-e.com/molecule.php?qn=417) | 47.75 | 0.24 |
| Glycyrrhiza uralensis Fisch | gancao | MOL000422 | [kaempferol](https://old.tcmsp-e.com/molecule.php?qn=422) | 41.88 | 0.24 |
| Glycyrrhiza uralensis Fisch | gancao | MOL004328 | [naringenin](https://old.tcmsp-e.com/molecule.php?qn=4328) | 59.29 | 0.21 |
| Glycyrrhiza uralensis Fisch | gancao | MOL004805 | [(2S)-2-[4-hydroxy-3-(3-methylbut-2-enyl)phenyl]-8,8-dimethyl-2,3-dihydropyrano[2,3-f]chromen-4-one](https://old.tcmsp-e.com/molecule.php?qn=4805) | 31.79 | 0.72 |
| Glycyrrhiza uralensis Fisch | gancao | MOL004806 | [euchrenone](https://old.tcmsp-e.com/molecule.php?qn=4806) | 30.29 | 0.57 |
| Glycyrrhiza uralensis Fisch | gancao | MOL004808 | [glyasperin B](https://old.tcmsp-e.com/molecule.php?qn=4808) | 65.22 | 0.44 |
| Glycyrrhiza uralensis Fisch | gancao | MOL004810 | [glyasperin F](https://old.tcmsp-e.com/molecule.php?qn=4810) | 75.84 | 0.54 |
| Glycyrrhiza uralensis Fisch | gancao | MOL004811 | [Glyasperin C](https://old.tcmsp-e.com/molecule.php?qn=4811) | 45.56 | 0.4 |
| Glycyrrhiza uralensis Fisch | gancao | MOL004814 | [Isotrifoliol](https://old.tcmsp-e.com/molecule.php?qn=4814) | 31.94 | 0.42 |
| Glycyrrhiza uralensis Fisch | gancao | MOL004815 | [(E)-1-(2,4-dihydroxyphenyl)-3-(2,2-dimethylchromen-6-yl)prop-2-en-1-one](https://old.tcmsp-e.com/molecule.php?qn=4815) | 39.62 | 0.35 |
| Glycyrrhiza uralensis Fisch | gancao | MOL004820 | [kanzonols W](https://old.tcmsp-e.com/molecule.php?qn=4820) | 50.48 | 0.52 |
| Glycyrrhiza uralensis Fisch | gancao | MOL004824 | [(2S)-6-(2,4-dihydroxyphenyl)-2-(2-hydroxypropan-2-yl)-4-methoxy-2,3-dihydrofuro[3,2-g]chromen-7-one](https://old.tcmsp-e.com/molecule.php?qn=4824) | 60.25 | 0.63 |
| Glycyrrhiza uralensis Fisch | gancao | MOL004827 | [Semilicoisoflavone B](https://old.tcmsp-e.com/molecule.php?qn=4827) | 48.78 | 0.55 |
| Glycyrrhiza uralensis Fisch | gancao | MOL004828 | [Glepidotin A](https://old.tcmsp-e.com/molecule.php?qn=4828) | 44.72 | 0.35 |
| Glycyrrhiza uralensis Fisch | gancao | MOL004829 | [Glepidotin B](https://old.tcmsp-e.com/molecule.php?qn=4829) | 64.46 | 0.34 |
| Glycyrrhiza uralensis Fisch | gancao | MOL004833 | [Phaseolinisoflavan](https://old.tcmsp-e.com/molecule.php?qn=4833) | 32.01 | 0.45 |
| Glycyrrhiza uralensis Fisch | gancao | MOL004835 | [Glypallichalcone](https://old.tcmsp-e.com/molecule.php?qn=4835) | 61.6 | 0.19 |
| Glycyrrhiza uralensis Fisch | gancao | MOL004838 | [8-(6-hydroxy-2-benzofuranyl)-2,2-dimethyl-5-chromenol](https://old.tcmsp-e.com/molecule.php?qn=4838) | 58.44 | 0.38 |
| Glycyrrhiza uralensis Fisch | gancao | MOL004841 | [Licochalcone B](https://old.tcmsp-e.com/molecule.php?qn=4841) | 76.76 | 0.19 |
| Glycyrrhiza uralensis Fisch | gancao | MOL004848 | [licochalcone G](https://old.tcmsp-e.com/molecule.php?qn=4848) | 49.25 | 0.32 |
| Glycyrrhiza uralensis Fisch | gancao | MOL004849 | [3-(2,4-dihydroxyphenyl)-8-(1,1-dimethylprop-2-enyl)-7-hydroxy-5-methox y-coumarin](https://old.tcmsp-e.com/molecule.php?qn=4849) | 59.62 | 0.43 |
| Glycyrrhiza uralensis Fisch | gancao | MOL004855 | [Licoricone](https://old.tcmsp-e.com/molecule.php?qn=4855) | 63.58 | 0.47 |
| Glycyrrhiza uralensis Fisch | gancao | MOL004856 | [Gancaonin A](https://old.tcmsp-e.com/molecule.php?qn=4856) | 51.08 | 0.4 |
| Glycyrrhiza uralensis Fisch | gancao | MOL004857 | [Gancaonin B](https://old.tcmsp-e.com/molecule.php?qn=4857) | 48.79 | 0.45 |
| Glycyrrhiza uralensis Fisch | gancao | MOL004860 | [licorice glycoside E](https://old.tcmsp-e.com/molecule.php?qn=4860) | 32.89 | 0.27 |
| Glycyrrhiza uralensis Fisch | gancao | MOL004863 | [3-(3,4-dihydroxyphenyl)-5,7-dihydroxy-8-(3-methylbut-2-enyl)chromone](https://old.tcmsp-e.com/molecule.php?qn=4863) | 66.37 | 0.41 |
| Glycyrrhiza uralensis Fisch | gancao | MOL004864 | [5,7-dihydroxy-3-(4-methoxyphenyl)-8-(3-methylbut-2-enyl)chromone](https://old.tcmsp-e.com/molecule.php?qn=4864) | 30.49 | 0.41 |
| Glycyrrhiza uralensis Fisch | gancao | MOL004866 | [2-(3,4-dihydroxyphenyl)-5,7-dihydroxy-6-(3-methylbut-2-enyl)chromone](https://old.tcmsp-e.com/molecule.php?qn=4866) | 44.15 | 0.41 |
| Glycyrrhiza uralensis Fisch | gancao | MOL004879 | [Glycyrin](https://old.tcmsp-e.com/molecule.php?qn=4879) | 52.61 | 0.47 |
| Glycyrrhiza uralensis Fisch | gancao | MOL004882 | [Licocoumarone](https://old.tcmsp-e.com/molecule.php?qn=4882) | 33.21 | 0.36 |
| Glycyrrhiza uralensis Fisch | gancao | MOL004883 | [Licoisoflavone](https://old.tcmsp-e.com/molecule.php?qn=4883) | 41.61 | 0.42 |
| Glycyrrhiza uralensis Fisch | gancao | MOL004884 | [Licoisoflavone B](https://old.tcmsp-e.com/molecule.php?qn=4884) | 38.93 | 0.55 |
| Glycyrrhiza uralensis Fisch | gancao | MOL004885 | [licoisoflavanone](https://old.tcmsp-e.com/molecule.php?qn=4885) | 52.47 | 0.54 |
| Glycyrrhiza uralensis Fisch | gancao | MOL004891 | [shinpterocarpin](https://old.tcmsp-e.com/molecule.php?qn=4891) | 80.3 | 0.73 |
| Glycyrrhiza uralensis Fisch | gancao | MOL004898 | [(E)-3-[3,4-dihydroxy-5-(3-methylbut-2-enyl)phenyl]-1-(2,4-dihydroxyphenyl)prop-2-en-1-one](https://old.tcmsp-e.com/molecule.php?qn=4898) | 46.27 | 0.31 |
| Glycyrrhiza uralensis Fisch | gancao | MOL004903 | [liquiritin](https://old.tcmsp-e.com/molecule.php?qn=4903) | 65.69 | 0.74 |
| Glycyrrhiza uralensis Fisch | gancao | MOL004904 | [licopyranocoumarin](https://old.tcmsp-e.com/molecule.php?qn=4904) | 80.36 | 0.65 |
| Glycyrrhiza uralensis Fisch | gancao | MOL004905 | [3,22-Dihydroxy-11-oxo-delta(12)-oleanene-27-alpha-methoxycarbonyl-29-oic acid](https://old.tcmsp-e.com/molecule.php?qn=4905) | 34.32 | 0.55 |
| Glycyrrhiza uralensis Fisch | gancao | MOL004907 | [Glyzaglabrin](https://old.tcmsp-e.com/molecule.php?qn=4907) | 61.07 | 0.35 |
| Glycyrrhiza uralensis Fisch | gancao | MOL004908 | [Glabridin](https://old.tcmsp-e.com/molecule.php?qn=4908) | 53.25 | 0.47 |
| Glycyrrhiza uralensis Fisch | gancao | MOL004910 | [Glabranin](https://old.tcmsp-e.com/molecule.php?qn=4910) | 52.9 | 0.31 |
| Glycyrrhiza uralensis Fisch | gancao | MOL004911 | [Glabrene](https://old.tcmsp-e.com/molecule.php?qn=4911) | 46.27 | 0.44 |
| Glycyrrhiza uralensis Fisch | gancao | MOL004912 | [Glabrone](https://old.tcmsp-e.com/molecule.php?qn=4912) | 52.51 | 0.5 |
| Glycyrrhiza uralensis Fisch | gancao | MOL004913 | [1,3-dihydroxy-9-methoxy-6-benzofurano[3,2-c]chromenone](https://old.tcmsp-e.com/molecule.php?qn=4913) | 48.14 | 0.43 |
| Glycyrrhiza uralensis Fisch | gancao | MOL004914 | [1,3-dihydroxy-8,9-dimethoxy-6-benzofurano[3,2-c]chromenone](https://old.tcmsp-e.com/molecule.php?qn=4914) | 62.9 | 0.53 |
| Glycyrrhiza uralensis Fisch | gancao | MOL004915 | [Eurycarpin A](https://old.tcmsp-e.com/molecule.php?qn=4915) | 43.28 | 0.37 |
| Glycyrrhiza uralensis Fisch | gancao | MOL004917 | [glycyroside](https://old.tcmsp-e.com/molecule.php?qn=4917) | 37.25 | 0.79 |
| Glycyrrhiza uralensis Fisch | gancao | MOL004924 | [(-)-Medicocarpin](https://old.tcmsp-e.com/molecule.php?qn=4924) | 40.99 | 0.95 |
| Glycyrrhiza uralensis Fisch | gancao | MOL004935 | [Sigmoidin-B](https://old.tcmsp-e.com/molecule.php?qn=4935) | 34.88 | 0.41 |
| Glycyrrhiza uralensis Fisch | gancao | MOL004941 | [(2R)-7-hydroxy-2-(4-hydroxyphenyl)chroman-4-one](https://old.tcmsp-e.com/molecule.php?qn=4941) | 71.12 | 0.18 |
| Glycyrrhiza uralensis Fisch | gancao | MOL004945 | [(2S)-7-hydroxy-2-(4-hydroxyphenyl)-8-(3-methylbut-2-enyl)chroman-4-one](https://old.tcmsp-e.com/molecule.php?qn=4945) | 36.57 | 0.32 |
| Glycyrrhiza uralensis Fisch | gancao | MOL004948 | [Isoglycyrol](https://old.tcmsp-e.com/molecule.php?qn=4948) | 44.7 | 0.84 |
| Glycyrrhiza uralensis Fisch | gancao | MOL004949 | [Isolicoflavonol](https://old.tcmsp-e.com/molecule.php?qn=4949) | 45.17 | 0.42 |
| Glycyrrhiza uralensis Fisch | gancao | MOL004957 | [HMO](https://old.tcmsp-e.com/molecule.php?qn=4957) | 38.37 | 0.21 |
| Glycyrrhiza uralensis Fisch | gancao | MOL004959 | [1-Methoxyphaseollidin](https://old.tcmsp-e.com/molecule.php?qn=4959) | 69.98 | 0.64 |
| Glycyrrhiza uralensis Fisch | gancao | MOL004961 | [Quercetin der.](https://old.tcmsp-e.com/molecule.php?qn=4961) | 46.45 | 0.33 |
| Glycyrrhiza uralensis Fisch | gancao | MOL004966 | [3'-Hydroxy-4'-O-Methylglabridin](https://old.tcmsp-e.com/molecule.php?qn=4966) | 43.71 | 0.57 |
| Glycyrrhiza uralensis Fisch | gancao | MOL000497 | [licochalcone a](https://old.tcmsp-e.com/molecule.php?qn=497) | 40.79 | 0.29 |
| Glycyrrhiza uralensis Fisch | gancao | MOL004974 | [3'-Methoxyglabridin](https://old.tcmsp-e.com/molecule.php?qn=4974) | 46.16 | 0.57 |
| Glycyrrhiza uralensis Fisch | gancao | MOL004978 | [2-[(3R)-8,8-dimethyl-3,4-dihydro-2H-pyrano[6,5-f]chromen-3-yl]-5-methoxyphenol](https://old.tcmsp-e.com/molecule.php?qn=4978) | 36.21 | 0.52 |
| Glycyrrhiza uralensis Fisch | gancao | MO L004980 | [Inflacoumarin A](https://old.tcmsp-e.com/molecule.php?qn=4980) | 39.71 | 0.33 |
| Glycyrrhiza uralensis Fisch | gancao | MOL004985 | [icos-5-enoic acid](https://old.tcmsp-e.com/molecule.php?qn=4985) | 30.7 | 0.2 |
| Glycyrrhiza uralensis Fisch | gancao | MOL004988 | [Kanzonol F](https://old.tcmsp-e.com/molecule.php?qn=4988) | 32.47 | 0.89 |
| Glycyrrhiza uralensis Fisch | gancao | MOL004989 | [6-prenylated eriodictyol](https://old.tcmsp-e.com/molecule.php?qn=4989) | 39.22 | 0.41 |
| Glycyrrhiza uralensis Fisch | gancao | MOL004990 | [7,2',4'-trihydroxy－5-methoxy-3－arylcoumarin](https://old.tcmsp-e.com/molecule.php?qn=4990) | 83.71 | 0.27 |
| Glycyrrhiza uralensis Fisch | gancao | MOL004991 | [7-Acetoxy-2-methylisoflavone](https://old.tcmsp-e.com/molecule.php?qn=4991) | 38.92 | 0.26 |
| Glycyrrhiza uralensis Fisch | gancao | MOL004993 | [8-prenylated eriodictyol](https://old.tcmsp-e.com/molecule.php?qn=4993) | 53.79 | 0.4 |
| Glycyrrhiza uralensis Fisch | gancao | MOL004996 | [gadelaidic acid](https://old.tcmsp-e.com/molecule.php?qn=4996) | 30.7 | 0.2 |
| Glycyrrhiza uralensis Fisch | gancao | MOL000500 | [Vestitol](https://old.tcmsp-e.com/molecule.php?qn=500) | 74.66 | 0.21 |
| Glycyrrhiza uralensis Fisch | gancao | MOL005000 | [Gancaonin G](https://old.tcmsp-e.com/molecule.php?qn=5000) | 60.44 | 0.39 |
| Glycyrrhiza uralensis Fisch | gancao | MOL005001 | [Gancaonin H](https://old.tcmsp-e.com/molecule.php?qn=5001) | 50.1 | 0.78 |
| Glycyrrhiza uralensis Fisch | gancao | MOL005003 | [Licoagrocarpin](https://old.tcmsp-e.com/molecule.php?qn=5003) | 58.81 | 0.58 |
| Glycyrrhiza uralensis Fisch | gancao | MOL005007 | [Glyasperins M](https://old.tcmsp-e.com/molecule.php?qn=5007) | 72.67 | 0.59 |
| Glycyrrhiza uralensis Fisch | gancao | MOL005008 | [Glycyrrhiza flavonol A](https://old.tcmsp-e.com/molecule.php?qn=5008) | 41.28 | 0.6 |
| Glycyrrhiza uralensis Fisch | gancao | MOL005012 | [Licoagroisoflavone](https://old.tcmsp-e.com/molecule.php?qn=5012) | 57.28 | 0.49 |
| Glycyrrhiza uralensis Fisch | gancao | MOL005013 | [18α-hydroxyglycyrrhetic acid](https://old.tcmsp-e.com/molecule.php?qn=5013) | 41.16 | 0.71 |
| Glycyrrhiza uralensis Fisch | gancao | MOL005016 | [Odoratin](https://old.tcmsp-e.com/molecule.php?qn=5016) | 49.95 | 0.3 |
| Glycyrrhiza uralensis Fisch | gancao | MOL005017 | [Phaseol](https://old.tcmsp-e.com/molecule.php?qn=5017) | 78.77 | 0.58 |
| Glycyrrhiza uralensis Fisch | gancao | MOL005018 | [Xambioona](https://old.tcmsp-e.com/molecule.php?qn=5018) | 54.85 | 0.87 |
| Glycyrrhiza uralensis Fisch | gancao | MOL005020 | [dehydroglyasperins C](https://old.tcmsp-e.com/molecule.php?qn=5020) | 53.82 | 0.37 |
| Glycyrrhiza uralensis Fisch | gancao | MOL000098 | [quercetin](https://old.tcmsp-e.com/molecule.php?qn=98) | 46.43 | 0.28 |
| Sophora flavescens Aiton | kushen | MOL001040 | [(2R)-5,7-dihydroxy-2-(4-hydroxyphenyl)chroman-4-one](https://old.tcmsp-e.com/molecule.php?qn=1040) | 42.36 | 0.21 |
| Sophora flavescens Aiton | kushen | MOL001484 | [Inermine](https://old.tcmsp-e.com/molecule.php?qn=1484) | 75.18 | 0.54 |
| Sophora flavescens Aiton | kushen | MOL003542 | [8-Isopentenyl-kaempferol](https://old.tcmsp-e.com/molecule.php?qn=3542) | 38.04 | 0.39 |
| Sophora flavescens Aiton | kushen | MOL003627 | [sophocarpine](https://old.tcmsp-e.com/molecule.php?qn=3627) | 64.26 | 0.25 |
| Sophora flavescens Aiton | kushen | MOL003648 | [Inermin](https://old.tcmsp-e.com/molecule.php?qn=3648) | 65.83 | 0.54 |
| Sophora flavescens Aiton | kushen | MOL003673 | [Wighteone](https://old.tcmsp-e.com/molecule.php?qn=3673) | 42.8 | 0.36 |
| Sophora flavescens Aiton | kushen | MOL003676 | [Sophoramine](https://old.tcmsp-e.com/molecule.php?qn=3676) | 42.16 | 0.25 |
| Sophora flavescens Aiton | kushen | MOL003680 | [sophoridine](https://old.tcmsp-e.com/molecule.php?qn=3680) | 60.07 | 0.25 |
| Sophora flavescens Aiton | kushen | MOL000392 | [formononetin](https://old.tcmsp-e.com/molecule.php?qn=392) | 69.67 | 0.21 |
| Sophora flavescens Aiton | kushen | MOL004580 | [cis-Dihydroquercetin](https://old.tcmsp-e.com/molecule.php?qn=4580) | 66.44 | 0.27 |
| Sophora flavescens Aiton | kushen | MOL004941 | [(2R)-7-hydroxy-2-(4-hydroxyphenyl)chroman-4-one](https://old.tcmsp-e.com/molecule.php?qn=4941) | 71.12 | 0.18 |
| Sophora flavescens Aiton | kushen | MOL005100 | [5,7-dihydroxy-2-(3-hydroxy-4-methoxyphenyl)chroman-4-one](https://old.tcmsp-e.com/molecule.php?qn=5100) | 47.74 | 0.27 |
| Sophora flavescens Aiton | kushen | MOL005944 | [matrine](https://old.tcmsp-e.com/molecule.php?qn=5944) | 63.77 | 0.25 |
| Sophora flavescens Aiton | kushen | MOL000006 | [luteolin](https://old.tcmsp-e.com/molecule.php?qn=6) | 36.16 | 0.25 |
| Sophora flavescens Aiton | kushen | MOL006561 | [(+)-14alpha-hydroxymatrine](https://old.tcmsp-e.com/molecule.php?qn=6561) | 35.73 | 0.29 |
| Sophora flavescens Aiton | kushen | MOL006562 | [(+)-7,11-dehydromatrine,(leontalbinine)](https://old.tcmsp-e.com/molecule.php?qn=6562) | 62.08 | 0.25 |
| Sophora flavescens Aiton | kushen | MOL006563 | [(+)-9alpha-hydroxymatrine](https://old.tcmsp-e.com/molecule.php?qn=6563) | 32.04 | 0.29 |
| Sophora flavescens Aiton | kushen | MOL006564 | [(+)-allomatrine](https://old.tcmsp-e.com/molecule.php?qn=6564) | 58.87 | 0.25 |
| Sophora flavescens Aiton | kushen | MOL006565 | [AIDS211310](https://old.tcmsp-e.com/molecule.php?qn=6565) | 68.68 | 0.25 |
| Sophora flavescens Aiton | kushen | MOL006566 | [(+)-lehmannine](https://old.tcmsp-e.com/molecule.php?qn=6566) | 58.34 | 0.25 |
| Sophora flavescens Aiton | kushen | MOL006568 | [isosophocarpine](https://old.tcmsp-e.com/molecule.php?qn=6568) | 61.57 | 0.25 |
| Sophora flavescens Aiton | kushen | MOL006569 | [(-)-14beta-hydroxymatrine](https://old.tcmsp-e.com/molecule.php?qn=6569) | 37.26 | 0.29 |
| Sophora flavescens Aiton | kushen | MOL006570 | [(-)-9alpha-hydroxysophoramine](https://old.tcmsp-e.com/molecule.php?qn=6570) | 35.23 | 0.29 |
| Sophora flavescens Aiton | kushen | MOL006571 | [anagyrine](https://old.tcmsp-e.com/molecule.php?qn=6571) | 62.01 | 0.24 |
| Sophora flavescens Aiton | kushen | MOL006572 | [1,4-diazaindan-type,alkaloid,flavascensine](https://old.tcmsp-e.com/molecule.php?qn=6572) | 34.64 | 0.24 |
| Sophora flavescens Aiton | kushen | MOL006573 | [13,14-dehydrosophoridine](https://old.tcmsp-e.com/molecule.php?qn=6573) | 65.34 | 0.25 |
| Sophora flavescens Aiton | kushen | MOL006582 | [5α,9α-dihydroxymatrine](https://old.tcmsp-e.com/molecule.php?qn=6582) | 40.93 | 0.32 |
| Sophora flavescens Aiton | kushen | MOL006583 | [7,11-dehydromatrine](https://old.tcmsp-e.com/molecule.php?qn=6583) | 44.43 | 0.25 |
| Sophora flavescens Aiton | kushen | MOL006596 | [Glyceollin](https://old.tcmsp-e.com/molecule.php?qn=6596) | 97.27 | 0.76 |
| Sophora flavescens Aiton | kushen | MOL003347 | [hyperforin](https://old.tcmsp-e.com/molecule.php?qn=3347) | 44.03 | 0.6 |
| Sophora flavescens Aiton | kushen | MOL006604 | [(2S)-7-hydroxy-2-(4-hydroxyphenyl)-5-methoxy-8-(3-methylbut-2-enyl)chroman-4-one](https://old.tcmsp-e.com/molecule.php?qn=6604) | 48.09 | 0.39 |
| Sophora flavescens Aiton | kushen | MOL006613 | [kushenin](https://old.tcmsp-e.com/molecule.php?qn=6613) | 47.62 | 0.38 |
| Sophora flavescens Aiton | kushen | MOL006619 | [kushenol J](https://old.tcmsp-e.com/molecule.php?qn=6619) | 51.39 | 0.74 |
| Sophora flavescens Aiton | kushen | MOL006620 | [kushenol J_qt](https://old.tcmsp-e.com/molecule.php?qn=6620) | 50.86 | 0.24 |
| Sophora flavescens Aiton | kushen | MOL006622 | [kushenol O](https://old.tcmsp-e.com/molecule.php?qn=6622) | 42.41 | 0.76 |
| Sophora flavescens Aiton | kushen | MOL006623 | [kushenol,t](https://old.tcmsp-e.com/molecule.php?qn=6623) | 51.28 | 0.64 |
| Sophora flavescens Aiton | kushen | MOL006626 | [leachianone,g](https://old.tcmsp-e.com/molecule.php?qn=6626) | 60.97 | 0.4 |
| Sophora flavescens Aiton | kushen | MOL006627 | [Lehmanine](https://old.tcmsp-e.com/molecule.php?qn=6627) | 62.23 | 0.25 |
| Sophora flavescens Aiton | kushen | MOL006628 | [(+)-Lupanine](https://old.tcmsp-e.com/molecule.php?qn=6628) | 52.71 | 0.24 |
| Sophora flavescens Aiton | kushen | MOL006630 | [Norartocarpetin](https://old.tcmsp-e.com/molecule.php?qn=6630) | 54.93 | 0.24 |
| Sophora flavescens Aiton | kushen | MOL000456 | [Phaseolin](https://old.tcmsp-e.com/molecule.php?qn=456) | 78.2 | 0.73 |
| Sophora flavescens Aiton | kushen | MOL006649 | [sophranol](https://old.tcmsp-e.com/molecule.php?qn=6649) | 55.42 | 0.28 |
| Sophora flavescens Aiton | kushen | MOL006650 | [(-)-Maackiain-3-O-glucosyl-6'-O-malonate](https://old.tcmsp-e.com/molecule.php?qn=6650) | 48.69 | 0.52 |
| Sophora flavescens Aiton | kushen | MOL006652 | [trifolrhizin](https://old.tcmsp-e.com/molecule.php?qn=6652) | 48.53 | 0.74 |
| Sophora flavescens Aiton | kushen | MOL000098 | [quercetin](https://old.tcmsp-e.com/molecule.php?qn=98) | 46.43 | 0.28 |
| Hedysarum polybotrys Hand.-Mazz. | hongqi | MOL011076 | (+)-Medicarpin | 60.46 | 0.34 |
| Hedysarum polybotrys Hand.-Mazz. | hongqi | MOL011078 | 3',7-dihydroxy-4'-methoxy-isoflavone | 50.7 | 0.24 |
| Hedysarum polybotrys Hand.-Mazz. | hongqi | MOL001792 | DFV | 32.76 | 0.18 |
| Hedysarum polybotrys Hand.-Mazz. | hongqi | MOL000358 | beta-sitosterol | 36.91 | 0.75 |
| Hedysarum polybotrys Hand.-Mazz. | hongqi | MOL000392 | formononetin | 69.67 | 0.21 |
| Hedysarum polybotrys Hand.-Mazz. | hongqi | MOL000417 | Calycosin | 47.75 | 0.24 |
| Hedysarum polybotrys Hand.-Mazz. | hongqi | MOL004328 | naringenin | 59.29 | 0.21 |
| Hedysarum polybotrys Hand.-Mazz. | hongqi | MOL000449 | Stigmasterol | 43.83 | 0.76 |
| Hedysarum polybotrys Hand.-Mazz. | hongqi | MOL004644 | Sainfuran | 79.91 | 0.23 |
| Hedysarum polybotrys Hand.-Mazz. | hongqi | MOL004941 | (2R)-7-hydroxy-2-(4-hydroxyphenyl)chroman-4-one | 71.12 | 0.18 |
| Hedysarum polybotrys Hand.-Mazz. | hongqi | MOL000500 | Vestitol | 74.66 | 0.21 |
| Hedysarum polybotrys Hand.-Mazz. | hongqi | MOL005575 | Gentiacaulein | 72.82 | 0.27 |
| Hedysarum polybotrys Hand.-Mazz. | hongqi | MOL008173 | daucosterol_qt | 36.91 | 0.75 |
| Hedysarum polybotrys Hand.-Mazz. | hongqi | MOL000098 | quercetin | 46.43 | 0.28 |
| Angelica sinensis (Oliv.) Diels | dangdui | MOL000358 | beta-sitosterol | 36.91 | 0.75 |
| Angelica sinensis (Oliv.) Diels | danggui | MOL000449 | Stigmasterol | 43.83 | 0.76 |
| Patrinia heterophylla | mutouhui | MOL000098 | quercetin | 46.43 | 0.28 |
| Curcuma phaeocaulis Valeton | ezhu | MOL000940 | bisdemethoxycurcumin | 77.38 | 0.26 |
| Curcuma phaeocaulis Valeton | eshu | MOL000906 | wenjine | 47.93 | 0.27 |
| Curcuma phaeocaulis Valeton | ezhu | MOL000296 | hederagenin | 36.91 | 0.75 |
